# Effect of Gonadotropin-Releasing Hormone Antagonist on Risk of Committing Child Sexual Abuse in Men With Pedophilic Disorder: A Randomized Clinical Trial

**DOI:** 10.1001/jamapsychiatry.2020.0440

**Published:** 2020-04-29

**Authors:** Valdemar Landgren, Kinda Malki, Matteo Bottai, Stefan Arver, Christoffer Rahm

**Affiliations:** 1 Institute of Neuroscience and Physiology, Gothenburg University, Gothenburg, Sweden; 2 Karolinska University Hospital, Stockholm, Sweden; 3 Institute of Environmental Medicine, Karolinska Institutet, Stockholm, Sweden; 4 Department of Medicine Huddinge, Karolinska Institutet, Stockholm, Sweden; 5 Centre for Psychiatry Research, Department of Clinical Neuroscience, Karolinska Institutet, Stockholm, Sweden; 6 Stockholm Health Care Services, Region Stockholm, Stockholm, Sweden

## Abstract

**Importance:**

Evidence-based treatments from randomized clinical trials for pedophilic disorder are lacking.

**Objective:**

To determine whether a gonadotropin-releasing hormone antagonist reduces dynamic risk factors for committing child sexual abuse.

**Design, Setting, and Participants:**

This academically initiated, double-blind, placebo-controlled, parallel-group, phase 2 randomized clinical trial was conducted at the ANOVA center in Stockholm, Sweden, from March 1, 2016, to April 30, 2019. Individuals who contacted PrevenTell, the national telephone helpline for unwanted sexuality, were recruited. Eligible participants were men seeking help aged 18 to 66 years with a pedophilic disorder diagnosis and no contraindications to the intervention. The primary end point was assessed by intent-to-treat analysis.

**Interventions:**

Randomization to receive either 2 subcutaneous injections of 120 mg of degarelix acetate or equal volume of placebo.

**Main Outcomes and Measures:**

The primary end point was the mean change between baseline and 2 weeks in the composite risk score of 5 domains of child sexual abuse ranging from 0 to 15 points; each domain could be rated from 0 to 3 points. Secondary end points included efficacy at 2 and 10 weeks as measured by the composite score, each risk domain, quality of life, self-reported effects, and adverse events.

**Results:**

A total of 52 male participants (mean [SD] age, 36 [12] years) were randomized to receive either degarelix (n = 25; with 1 withdrawal) or placebo (n = 26). At 2 weeks, the composite risk score decreased from 7.4 to 4.4 for participants in the degarelix group and from 7.8 to 6.6 for the placebo group, a mean between-group difference of –1.8 (95% CI, –3.2 to –0.5; *P* = .01). A decrease was seen in the composite score at 10 weeks (−2.2 [95% CI, −3.6 to −0.7]) as well as in the domains of pedophilic disorder (2 weeks: −0.7 [95% CI, −1.4 to 0.0]; 10 weeks: −1.1 [95% CI, −1.8 to −0.4]) and sexual preoccupation (2 weeks: −0.7 [95% CI, −1.2 to −0.3]; 10 weeks: −0.8 [95% CI, −1.3 to −0.3]) in the degarelix group compared with the placebo group. No difference was seen for the domains of self-rated risk (2 weeks: −0.4 [95% CI, −0.9 to 0.1]; 10 weeks: −0.5 [95% CI, −1 to 0.0]), low empathy (2 weeks: 0.2 [95% CI, −0.3 to 0.6]; 10 weeks: 0.2 [95% CI, −0.2 to 0.6]), and impaired self-regulation (2 weeks: −0.0 [95% CI, −0.7 to 0.6]; 10 weeks: 0.1 [95% CI, −0.5 to 0.8]), or quality of life (EuroQol 5 Dimensions questionnaire index score, 2 weeks: 0.06 [95% CI, −0.00 to 0.12], and 10 weeks: 0.04; 95% CI, −0.02 to 0.10; EuroQol visual analog scale, 2 weeks: 0.6 [95% CI, −9.7 to 10.9], and 10 weeks: 4.2 [95% CI, −6.0 to 14.4]). Two hospitalizations occurred from increased suicidal ideation, and more injection site reactions (degarelix: 22 of 25 [88%]; placebo: 1 of 26 [4%]) and hepatobiliary enzyme level elevations were reported by participants who received degarelix (degarelix: 11 of 25 [44%]; placebo: 2 of 26 [8%]). Among the 26 participants randomized to receive degarelix, 20 (77%) experienced positive effects (eg, improved attitude or behavior) on sexuality and 23 (89%) reported adverse effects on the body.

**Conclusion and Relevance:**

This trial found that degarelix reduced the risk score for committing child sexual abuse in men with pedophilic disorder 2 weeks after initial injection, suggesting use of the drug as a rapid-onset treatment option. Further studies are warranted into the effects and long-term adverse effects of hormone deficiency.

**Trial Registration:**

EU Clinical Trials Register Identifier: 2014-000647-32

## Key Points

**Question:**

Can a gonadotropin-releasing hormone antagonist rapidly reduce the risk for committing child sexual abuse in men with pedophilic disorder who are seeking help?

**Findings:**

In this randomized clinical trial of 52 men with pedophilic disorder, treatment with degarelix statistically significantly reduced the risk for committing child sexual abuse 2 weeks after the initial injection.

**Meaning:**

This finding suggests that degarelix may serve as a rapid-onset, risk-reducing medication for men with pedophilic disorder.

## Teaser

This randomized clinical trial evaluates the immediate efficacy of testosterone-suppressing medication in reducing the risk scores in 5 domains of child sexual abuse among men with pedophilic disorder.

## Introduction

Child sexual abuse affects 1 in 5 girls and 1 in 10 boys worldwide.^1^ It is accompanied by adverse psychosocial outcome across the life span.^1,2^ Preventive measures have been advocated,^3^ but to date evidence for interventions has been limited.^4,5^

The estimated proportion of child sexual offenders who are prosecuted is 1%.^1,4^ Of those who were prosecuted, up to 95% were first-time offenders^6^ and half of them had pedophilic disorder,^7^ defined as recurrent sexual attraction to prepubescent children associated with distress or negative consequences. Studies have found that not all men with pedophilic disorder commit a sexual offense, but those who do generally report struggling with their sexual urges for 10 years before committing a sexual crime.^6,8^ Consequently, an opportunity for prevention exists in treating high-risk individuals without prior convictions. Effective treatment could prevent child sexual abuse and reduce psychosocial stress for the individual with pedophilic disorder.

Currently recommended interventions include psychotherapy and antidepressants as well as testosterone-suppressing medications for high-risk individuals.^9^ Opinions about treatment are vehement. In many countries, an informed consent procedure is required for chemical castration. However, chemical castration is used as a compulsory legal sentence for child sexual offenders in some jurisdictions in the US, Asia, and Europe but is prohibited in other countries owing to ethical concerns of coercion and uncertain efficacy.^10,11^

Because they lower testosterone through receptor desensitization, gonadotropin-releasing hormone agonists are considered effective in reducing paraphilic symptoms.^12^ However, the use of these agonists is limited to supervised correctional settings because of the lack of randomized clinical trials; their metabolic adverse effects; and the initial flare-up of testosterone, which may be associated with increased aggression and libido that require concurrent antiandrogen medication.^9^ Experience from the Swedish helpline PrevenTell suggests a need for both rapid-acting short-term treatment (eg, in critical phases of the disorder to quickly control sexual impulses or reduce a high degree of struggling) and long-term treatment. Degarelix acetate is a gonadotropin-releasing hormone antagonist that was approved by the US Food and Drug Administration in 2008 for treating advanced prostate cancer. Degarelix decreases testosterone to castration levels within 3 days without testosterone flare^13^; therefore, the drug could serve as a rapid-onset treatment for individuals seeking help in outpatient settings.^9,14,15^

For individuals without a prior conviction, no validated measures of risk exist. Therefore, the PRIOTAB (Pedophilia at Risk–Investigations of Treatment and Biomarkers) project, of which this present trial is a part, included the construction of a composite score based on previous observational studies of dynamic (ie, potentially changeable over time) risk factors for sexual offense recidivism. One such risk factor is deviant sexual interest (eg, pedophilic disorder).^16^ Other factors are sexual preoccupation, impaired self-regulation, and low empathy.^16^ Because these 4 factors are all possibly mitigated by testosterone suppression, we believed that degarelix could have a risk-reducing effect.^17,18,19,20^ Bearing in mind the challenges associated with conducting a trial in a hard-to-reach population along with the issues of tolerability of previous therapies, we performed ancillary interviews of self-reported experiences of treatment. We regarded using sexually arousing material and measuring participants’ penile responses as too intrusive.

We hypothesized that men with pedophilic disorder who were randomized to receive degarelix compared with placebo would have a substantial reduction in risk of committing child sexual abuse after 2 weeks. Furthermore, we hypothesized that degarelix would be sufficiently tolerated by the participants and thus would be a safe and effective rapid-onset treatment option.

## Methods

This randomized clinical trial was approved by the Swedish Central Ethical Review Board and the Swedish Medical Products Agency (trial protocol in Supplement 1). All participants signed an informed consent agreement and were offered treatment after the study. To ensure adherence to good clinical practice, the Karolinska Trial Alliance, a research center that supports clinical trials, provided independent monitoring and recommended a longer follow-up period to monitor the safety of the intervention; hence, a second follow-up visit with the same outcome measures was conducted at 10 weeks. The study was conducted without any kind of collaboration with the pharmaceutical industry. This trial followed the Consolidated Standards of Reporting Trials (CONSORT) reporting guideline.

### Trial Design and Participants

This academically initiated, double-blind, placebo-controlled, parallel-group phase 2 trial with balanced randomization (1:1) was conducted at the ANOVA center, a highly specialized center for andrology and sexual medicine in Stockholm, Sweden, from March 1, 2016, to April 30, 2019. The trial was conducted conjointly with a case-control study, for which participants underwent magnetic resonance imaging and additional testing at the same time and venue. The ANOVA center hosts PrevenTell, a national telephone helpline for self-identified unwanted sexuality, through which trial participants were recruited. In Sweden, the legal obligation to report suspected child abuse supersedes professional confidentiality, which may make individuals with pedophilic disorder reluctant to seek help. To minimize compromises in recruitment rates and to attain authenticity in self-reports, we provided participants a temporary identification number. This identification number kept participants anonymous to trial assessors (S.A., C.R.), although they were still informed of the professionals’ obligation, according to the Swedish Social Services Act, to send a notification of concern to the authorities when they suspect a child is at risk of abuse or maltreatment and the legal possibility of reporting to the police suspected perpetrators of crimes against children.

Participants were recruited from March 1, 2016, to January 31, 2019. A flow diagram of the participants through each stage of the trial is shown in the Figure. Eligible participants were help-seeking, self-identified men aged 18 to 66 years with a pedophilic disorder diagnosis, as defined in the *Diagnostic and Statistical Manual of Mental Disorders* (Fifth Edition). A full list of inclusion and exclusion criteria is provided in eAppendix 1 in Supplement 2. Participants recruited through PrevenTell were offered transportation costs for study visits and financial compensation of Sk 1000 (Swedish krona) (US $100 before taxation) after study completion.

**Figure.**
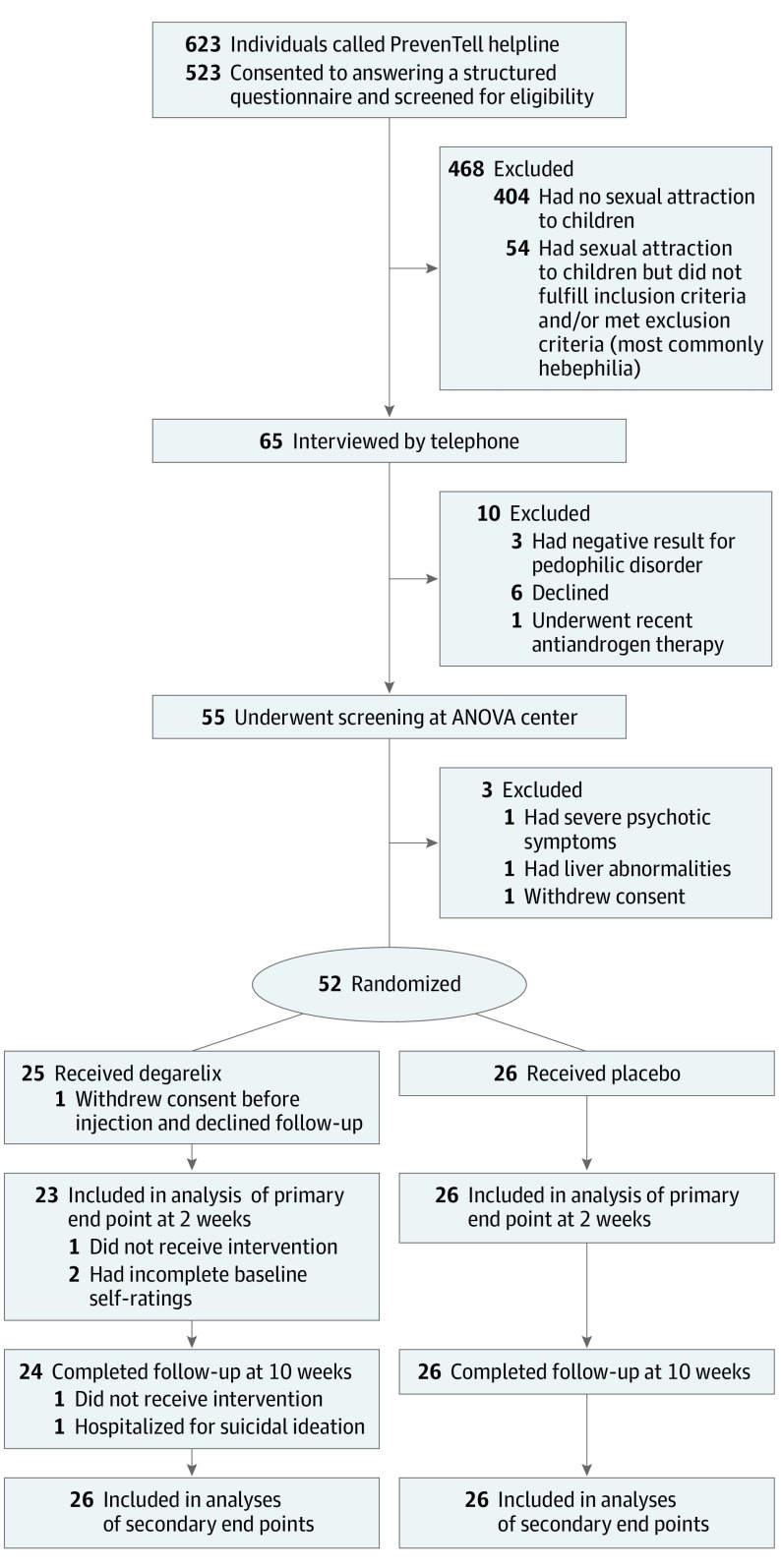
CONSORT Diagram

### Outcome Measures

The primary end point was the mean change in composite risk score (range, 0-15 points) between baseline and 2 weeks after randomization. The composite risk score consisted of 5 domains: the 4 empirically derived dynamic risk factors (pedophilic disorder, sexual preoccupation, impaired self-regulation, and low empathy)^16^ and self-rated risk, each of which could be rated from 0 to 3 points (Table 1; eAppendix 1 in Supplement 2). Each domain was weighted equally in the score. Three risk groups were prespecified (risk class 1: 0-5 points; risk class 2: 6-10 points; risk class 3: 11-15 points).

**Table 1.** Composite Score of Dynamic Risk for Committing Child Sexual Abuse^a^

Risk domain	Score definition			
	0	1	2	3
Pedophilic disorder^b^	No pedophilic attraction	Pedophilic attraction	Pedophilic attraction + distress or negative consequences	Pedophilic attraction + distress + negative consequences
Sexual preoccupation^c^	Hyposexual according to the SDI	Not hyposexual according to the SDI	Not hyposexual + hypersexual according to the HBI; no ongoing abusive behavior according to the SChiMRA-B	Not hyposexual + hypersexual + ongoing abusive behavior
Impaired self-regulation^d^	Normal CCPT II result	1 Abnormal CCPT II domain out of the inattention, impulsivity, and vigilance domains	2 Abnormal CCPT II domains of the inattention, impulsivity, and vigilance domains	All 3 abnormal CCPT II domains of inattention, impulsivity, and vigilance
Low empathy^e^	No abnormality	1 of RAADS-14 mentalizing domain >10, RMET <22, or current antisocial behavior according to the MINI	2 of RAADS-14 mentalizing domain >10, RMET <22, or current antisocial behavior	RAADS-14 mentalizing domain >10, + RMET<22, + current antisocial behavior
Self-rated risk^f^	Normal SChiMRA-A result	1 Domain of SChiMRA-A watch, socialize, or interact domain	2 Domains of SChiMRA-A watch, socialize, or interact domain	SChiMRA-A watch + socialize + interact domains

Abbreviations: CCPT II, Conners Continuous Performance Test; HBI, Hypersexual Behavior Inventory (score range: 19-95, with higher scores indicating more severe hypersexual behavior); MINI, Mini International Neuropsychiatric Interview; RAADS-14, Ritvo Autism and Asperger Diagnostic Scale, 14 Screen (score range: 0-42, of which the mentalizing domain ranges from 0 to 21, with higher scores indicating more autistic features); RMET, Reading the Mind in the Eyes Test (score range: 0-36, with higher scores indicating better capacity for emotion recognition); SChiMRA-A, Sexual Child Molestation Risk Assessment part A; SDI, Sexual Desire Inventory (score range: 12-104, with higher scores indicating increased sexual desire); VAS, visual analog scale (score range: 0%-100%, with higher scores indicating increased self-rated risk).

^a^ For a full description of the composite risk score, see eAppendix 1 in Supplement 2.

^b^ The 3 criteria for pedophilic disorder according to the *DSM-5* are pedophilic interest, significant distress, and significant negative consequences.

^c^ *Hyposexuality* is defined as a score of <45 on the SDI.^21^ *Hypersexuality* is defined as an HBI^22^ score of ≥53. The SChiMRA-B (eAppendix 1 in Supplement 2) assesses self-reported frequency of sexually abusive behavior in the past week (never, several days, more than half of days, or almost every day) regarding watching of, socializing with, and sexual interaction with children, in which occurrence of any sort is scored as positive.

^d^ CCPT II^23^ for inattention, impulsivity, and vigilance.

^e^ Self-ratings on the mentalization domains of the RAADS-14,^24^ RMET^25^ scores, and current antisocial behavior as reported in the MINI.^26^

^f^ The SChiMRA-A (eAppendix 1 in Supplement 2) consists of VAS ratings to the question, “How likely is it that you would do any of the following, if there was an easy way to do it without being caught?” regarding watching of, socializing with, and sexual interaction with children. A rating of 40% or higher on the VAS was interpreted as a substantial risk.

We used the prespecified analysis for the primary end point. This analysis included only participants with complete data at baseline and 2 weeks after injection.

The secondary end point analyses excluded no data. Secondary end points were efficacy at 2 and 10 weeks in terms of reduction in the composite risk score and its 5 domains, efficacy in participants in risk class 3 (11-15 points), quality of life, adverse events, and self-reported effects. Quality of life was measured at all time points, using the EuroQol 5 Dimensions (EQ-5D) questionnaire, which consists of a preference-based health status measure convertible into index scores ranging from 0 to 1 (with the higher score indicating better health status), and a EuroQol visual analog scale (EQ-VAS) of 0 to 100, by which higher scores represent better health status.^27,28^

Participants were given a study diary and instructed to note any adverse events between assessments and to call the study nurse if they perceived serious harm from treatment. At follow-up, physical adverse events were registered by the study nurse using both participant responses to open-ended questions about current health status and notes in the diary. Adverse events were documented by the assessing psychiatrist (C.R.) also, and all events were coded according to the MedDRA (Medical Dictionary for Drug Regulatory Affairs).^29^ Self-reported experiences were collected in a structured interview, including open-ended questions about the positive (eg, improved attitudes or behaviors) and negative (eg, adverse events) effects of the injection and willingness to maintain the experienced effects with further injections. Interviews were analyzed using qualitative descriptive content analysis, as described in eAppendix 2 in Supplement 2.^30^ Neither the assessors nor the participants were aware of treatment randomization at the time of the interviews or qualitative analysis.

### Trial Intervention

On the first visit, participants underwent eligibility and baseline evaluations and received the study drug (2 subcutaneous injections of 120 mg degarelix acetate or equal volume of placebo). A computer-generated sequence with permuted block randomization was provided by the Karolinska Trial Alliance. The randomization sequence was concealed from one of us (C.R. and S.A.) who enrolled and assessed participants and then transferred onto cards placed in sequentially numbered, opaque envelopes stored in a locked cabinet in the dispensary and accessible to only 1 independent study nurse, who in turn informed the nurse responsible for drug administration. A detailed description of the injection procedure is provided in eAppendix 1 in Supplement 2. Except for the study nurse who was responsible for treatment randomization and who also registered the physical adverse effects, all of the assessors involved in the repeated outcome assessments were blinded to the treatment allocation.

### Statistical Analysis

The sample size of this trial was determined from the experience of similar, previously published studies of biomarkers of treatment response. We calculated that the planned sample size of 60 would give a power greater than 90% to detect a clinically significant difference in the primary end point. We had no prespecified expected dropout rate.

The primary end point was assessed by intent-to-treat analysis, using the unequal-variance, 2-sided *t* test at interim analysis and at 2 weeks. To preserve the overall significance level of the test at .05, we chose a 2-sided *P* = .0294 (exact value given per the prespecified statistical plan).^31^ Participants for whom the score change could not be calculated (ie, missing values for the risk score at baseline or 2 weeks) were excluded from analysis of the primary end point under the assumption that the missing-data generating process was at random. In addition, we evaluated the trajectories of the secondary end points in the 2 treatment groups at the 3 visits, including all randomized participants. The mean of the numeric secondary end points was estimated using linear random-effects regression models, including the treatment indicator (binary covariate), indicator variables for the 2 follow-up visits (binary), and the 2 interaction terms between the treatment indicator and the 2 visit indicators (binary). The models also included a participant-specific random intercept, which was assumed to follow a normal distribution. The random intercept was included to consider the potential dependence in the repeated observations on each participant.

We examined the differences in the time trajectories between the treatment groups by testing the composite hypothesis that the interaction terms were jointly equal to zero using Wald-type tests. We used the estimates from the models to estimate the mean of the numeric end points. The SEs used to calculate their CIs were obtained with the delta method. All statistical analyses were performed using Stata, version 15 (StataCorp LLC).

When 20 participants had completed the study, the planned interim analysis of efficacy for the primary end point and a review of the safety of the treatment were performed by the sponsor (S.A.), who could find no statistically significant differences between the groups. Because no evidence of substantial harm from active treatment was found that would motivate the premature termination of the study, the study was permitted to continue with the planned sample size of 60.

## Results

### Participants and Intervention

Of the 52 male participants (mean [SD] age, 36 [12] years), 26 (50%; with 1 withdrawal) were randomized to receive degarelix and 26 (50%) to placebo. Of these 52 participants, 39 (75%) lived in urban areas of more than 50 000 inhabitants. The travel distance from home to the study center was more than 100 km for 28 participants (54%), 6 of whom (12%) traveled more than 400 km. Participant characteristics are described in Table 2. Minor differences in demographic and clinical characteristics were observed between the 2 treatment groups at baseline, but the median (interquartile range [IQR]) composite risk score was about the same (degarelix: 7.5 [6.0-8.0] vs placebo: 8 [6.8-9.0]) (eTable 1 in Supplement 2). A high prevalence of depression (35%) was found at baseline. All participants were in either risk class 1 (n = 6) or risk class 2 (n = 44), but 3 individuals in each treatment group had a baseline score of 10 points and were therefore analyzed as a high-risk subgroup. The median (IQR) number of days from baseline to visits was 15 (14-18) days at 2 weeks and 73 (73-74) days at 10 weeks for those in the degarelix group and 14 (14-14) days at 2 weeks and 73 (73-77) days at 10 weeks for those in the placebo group. The mean (SD) castration levels for serum testosterone were ascertained at 2 weeks (0.7 [0.2] nmol/L) and 10 weeks (0.6 [0.2] nmol/L) in all participants randomized to receive degarelix (eTable 4 in Supplement 2).

**Table 2.** Demographic and Clinical Characteristics of Participants at Baseline

Variable	No. (%)	
	Degarelix acetate (n = 26)	Placebo (n = 26)
Demographic characteristics		
Age, median (IQR) [range], y	36 (25-39) [19-54]	35 (28-47) [18-66]
Highest completed educational level		
Primary school, 9 y	2 (8)	3 (12)
Secondary education, 12 y	12 (46)	13 (50)
Postsecondary education	12 (46)	10 (38)
Unemployed	12 (46)	9 (35)
Living status		
Caregiver of child	7 (27)	12 (46)
Living without partner	17 (65)	17 (65)
Ever lived with a partner ≥2 y	10 (38)	15 (58)
Self-reported prior criminal conviction		
Noncontact sexual offense	4 (15)	4 (15)
Contact sexual offense	2 (8)	3(12)
Nonsexual offense	2 (8)	5 (19)
Sexuality		
Attraction primarily to boys	4 (15)	4 (15)
Attraction primarily to girls	19 (73)	21 (81)
Attraction to boys and girls	3 (12)	1 (4)
Attraction exclusively to prepubescent children	2 (8)	9 (35)
Age at discovery of attraction to minors, median (IQR), y^a^	16 (14-23)	16 (13-25)
Psychiatric characteristics^b^		
Any psychiatric disorder^c^	18 (69)	25 (96)
Ongoing depression	7 (27)	12 (46)
MADRS-S score in patients with depression, median (IQR)	25 (24-29)	24 (21-27)
Current psychotic symptoms	0	2 (8)
Previous manic episode	0	1 (4)
Hazardous drug or alcohol use	4 (17)	11 (42)
Full-scale IQ, median (IQR)	103 (93-118)	99 (96-116)
Psychoactive medication		
Antidepressants	7 (27)	8 (31)
Other^d^	7 (27)	5 (19)
Testosterone, median (IQR), nmol/L	16.5 (11-18.5)	14.0 (10.8-18.3)
Composite risk score, median (IQR)^e^	7.5 (6.0-8.0)	8 (6.8-9.0)

Abbreviations: ASRS, Adult ADHD (attention-deficit/hyperactivity disorder) Self-Report Scale screener (with a minimum of 4 of 6 questions rated above cutoff indicating ADHD); IQR, interquartile range; MADRS, Montgomery-Åsberg Depression Rating Scale (score range, 0-48 points, with higher scores indicating increased depression severity); MINI, Mini International Neuropsychiatric Interview); RAADS-14, Ritvo Autism and Asperger Diagnostic Scale, 14 Screen.

^a^ Two participants in the placebo group and 2 in the degarelix group stated that the attraction had “always been present.”

^b^ Based on the Wechsler Adult Intelligence Scale 4 (with the score of 100 indicating the mean intelligence of the population), MINI, MADRS-S self-rating version,^32^ Alcohol Use Disorder Identification Test (score of ≥8; score range: 0-40, with higher scores indicating more alcohol abuse) and the Drug Use Disorder Identification Test (score of ≥3; score range: 0-44, with higher scores indicating more drug abuse),^33,34^ ASRS Self-Report Scale screener,^35^ and RAADS-14.

^c^ As indicated by MINI, ASRS, and RAADS-14 scores.

^d^ Other medications included sleep medications, antihistamines, stimulants, and mood stabilizers.

^e^ Composite score ranged from 0 to 15 points, with higher scores indicating higher risk. In an age-matched comparison sample of men (n = 55), the median (IQR) composite risk score was 2 (1-2.5).

Because the retention rate of participants at 10 weeks was high (96%; n = 50), analysis of secondary end points was considered feasible without reaching the planned sample size of 60. This finding, along with a temporarily slow inclusion rate, ended the trial enrollment after randomization of 52 participants, a decision we made before unblinding.

### Outcomes

Primary and secondary end points of efficacy in reducing the composite risk score at 2 and 10 weeks differed substantially, decreasing from 7.4 to 4.4 points for participants in the degarelix group and from 7.8 to 6.6 points for the placebo group (mean difference: –1.8 [95% CI, –3.2 to –0.5]; *P* = .01) (Table 3). In the regression models of secondary end points, statistically significant differences were observed in composite risk scores (2 weeks: –1.8 [95% CI, –3.2 to –0.5]; 10 weeks: −2.2 [95% CI, −3.6 to −0.7]) (eFigure 1 and eFigure 2 in Supplement 2) and high-risk group (2 weeks: −3.3 [95% CI, −7.9 to 1.2]; 10 weeks: −6 [95% CI, −10.6 to−1.4]), between the pedophilic disorder (2 weeks: −0.7 [95% CI, −1.4 to 0.0]; 10 weeks: −1.1 [95% CI, −1.8 to −0.4]) and sexual preoccupation (2 weeks: −0.7 [95% CI, −1.2 to −0.3]; 10 weeks: −0.8 [95% CI, −1.3 to −0.3]) (eFigure 3 and eFigure 4 in Supplement 2) domains. No statistically significant differences in scores were seen in the domains of low empathy (2 weeks: 0.2 [95% CI, −0.3 to 0.6]; 10 weeks: 0.2 [95% CI, −0.2 to 0.6]), impaired self-regulation (2 weeks: −0.0 [95% CI, −0.7 to 0.6]; 10 weeks: 0.1 [95% CI, −0.5 to 0.8]), and self-rated risk (2 weeks: −0.4 [95% CI, −0.9 to 0.1]; 10 weeks: −0.5 [95% CI, −1 to 0.0]) or in either measure of quality of life (EQ-5D index score, 2 weeks: 0.06 [95% CI, −0.00 to 0.12], and 10 weeks: 0.04 [95% CI, −0.02 to 0.10]; EQ-VAS, 2 weeks: 0.6 [95% CI, −9.7 to 10.9], and 10 weeks: 4.2 [95% CI, −6.0 to 14.4]) (eTable 6 in Supplement 2). Themes and categories from the ancillary interviews are displayed in Table 4. In the degarelix group, positive attitudes toward sexuality (20 of 26 [77%]) and adverse effects on the body (23 of 26 [89%]) were the most common self-reported experiences. Analysis of specific participant quotes is provided in eAppendix 2 in Supplement 2.

**Table 3.** Primary and Secondary End Points

End points	Degarelix acetate			Placebo			Difference (95% CI)^a^			*P* value^b^
	Baseline (n = 24)	2 wk (n = 25)	10 wk (n = 24)	Baseline (n = 26)	2 wk (n = 26)	10 wk (n = 26)	Baseline	2 wk	10 wk	
Primary end points, mean (SE)^c^										
Composite risk score	7.4 (0.3)	4.4 (0.6)	NA	7.8 (0.3	6.6 (0.5)	NA	NA	−1.8 (−3.2 to −0.5)	NA	.01
Secondary end points [all data], mean (delta method SE)										
Composite risk score	7.3 (0.5)	4.4 (0.5)	3.6 (0.5)	7.8 (0.5)	6.6 (0.5)	6.2 (0.5)	−0.5 (−1.8 to 0.8)	−1.8 (−3.2 to −0.3)	−2.2 (−3.6 to −0.7)	.01
High risk group [ = 10 points] (n = 3 + 3)	10 (1.2)	6.7 (1.2)	2.3 (1.2)	10 (1.2)	10 (1.2)	8.3 (1.2)	0.0 (−3.3 to 3.3)	−3.3 (−7.9 to 1.2)	−6 (−10.6 to −1.4)	.04
Risk domain										
Pedophilic disorder	2.4 (0.2)	0.9 (0.2)	0.8 (0.2)	2.6 (0.2)	1.8 (0.2)	2.1 (0.2)	−0.2 (−0.7 to 0.3)	−0.7 (−1.4 to 0.0)	−1.1 (−1.8 to −0.4)	.01
Sexual preoccupation	1.6 (0.1)	0.7 (0.1)	0.4 (0.1)	1.6 (0.1)	1.4 (0.1)	1.2 (0.1)	0.0 (−0.4 to 0.4)	−0.7 (−1.2 to −0.3)	−0.8 (−1.3 to −0.3)	.001
Impaired self-regulation	1.5 (0.2)	1.2 (0.2)	1.4 (0.2)	1.4 (0.2)	1.2 (0.2)	1.2 (0.2)	0.0 (−0.5 to 0.6)	−0.0 (−0.7 to 0.6)	0.1 (−0.5 to 0.8)	.82
Low empathy	0.8 (0.2)	1.1 (0.2)	0.8 (0.2)	1 (0.2)	1.1 (0.2)	0.8 (0.2)	−0.2 (−0.6 to 0.3)	0.2 (−0.3 to 0.6)	0.2 (−0.2 to 0.6)	.61
Self-rated risk	1.0 (0.2)	0.5 (0.2)	0.2 (0.2)	1.2 (0.2)	1.1 (0.2)	0.9 (0.2)	−0.2 (−0.8 to 0.3)	−0.4 (−0.9 to 0.1)	−0.5 (−1 to 0.0)	.16
Quality of life										
EQ-5D index score	0.79 (0.03)	0.83 (0.03)	0.82 (0.03)	0.86 (0.0.3)	0.83 (0.03)	0.85 (0.03)	−0.06 (−1.37 to 0.01)	0.06 (−0.00 to 0.12)	0.04 (−0.02 to 0.10)	.16
EQ-VAS	59.8 (4.5)	59.0 (4.4)	61.3 (4.5)	60.5 (4.5)	59.1 (4.5)	57.8 (4.5)	−0.6 (−13.0 to 11.7)	0.6 (−9.7 to 10.9)	4.2 (−6.0 to 14.4)	.68

Abbreviations: EQ-5D, EuroQol 5 Dimensions questionnaire (score range, 0-1, with a higher score indicating better health status); EQ-VAS, EuroQol visual analog scale questionnaire (score range, 0-100, with higher scores indicating better health status); NA, not applicable.

^a^ Differences indicate the status of the participants randomized to receive degarelix.

^b^ *P* value is presented for a 2-sample *t* test with unequal variances for the primary end point and for a test for different time trajectories between groups in the random-effects regression models for all secondary end points.

^c^ Complete data for calculating the difference (baseline and 2 weeks) were available for 23 participants randomized to degarelix and 26 participants randomized to placebo (Figure).

**Table 4.** Questions and Categories from Self-reported Treatment Experiences^a^

Questions/Categories^b^	No. (%)	
	Degarelix acetate (n = 26)	Placebo (n = 26)
“What positive/negative effects do you experience from the injection?”^c^		
Positive effects of treatment		
Positive effects on sexuality	20 (77)	11 (42)
Improved mental health	4 (15)	1 (4)
Changed perspective	4 (15)	3 (12)
Improved cognitive ability	1 (4)	0
Improved self-control	1 (4)	1 (4)
Positive effects on relationship	1 (4)	0
Improved physical health	0	1 (4)
Negative effects of treatment		
Negative effects on body	23 (89)	11 (42)
Negative effects on sexuality	11 (42)	4 (15)
Relationship problems	4 (15)	2 (8)
Mental health issues	1 (4)	4 (15)
Decreased cognitive ability	1 (4)	1 (4)
Negative emotions	1 (4)	0
Negative effects on work	1 (4)	0
“Would you like a repeated injection maintaining the effects for another 10 weeks? Please explain your answer.”^d^		
Reasons for continuing treatment		
Overall positive effects	3 (12)	0
Positive emotions	2 (8)	0
Positive effects on sexuality	1 (4)	0
It’s necessary	1 (4)	0
Legal matter	0	1 (4)
To achieve effect	0	1 (4)
Positive effect on relationship	0	1 (4)
Reasons for discontinuing treatment		
Negative effects on sexuality	2 (8)	0
Negative effects on body	2 (8)	1 (4)
Achieved effect	2 (8)	0
Cautiousness	1 (4)	0
No effect	1(4)	9 (35)
Attitudes about treatment		
Positive attitude	6 (23)	1 (4)

^a^ Participants were interviewed with open-ended questions. Themes and categories were abstracted from the answers through qualitative content analysis (eAppendix 2 in Supplement 2).

^b^ Categories are displayed in descending order of frequency in the degarelix group.

^c^ Combining answers from 2 and 10 weeks. The question was asked in 2 steps: (1) “What positive effects do you experience from the injection?” and then (2) “What negative effects do you experience from the injection?” Positive effects refers to improvement in attitudes, behaviors, thinking, and relationships. Negative effects refers to adverse events.

^d^ Question asked only at 10 weeks. Three themes were abstracted. In the degarelix vs placebo groups, 15 vs 9 responded “yes” and 9 vs 17 responded “no.”

Post hoc analyses revealed that 15 of the 26 participants (58%) in the degarelix group and 3 of the 26 participants (12%) in the placebo group denied sexual attraction to minors at 10 weeks.

### Adverse Events

No serious adverse events occurred in the placebo group. A serious adverse event of increased suicidal ideation was reported by 2 of 25 participants (8%) in the degarelix group, which led to hospitalization. One participant reported the event at 10 weeks, when his condition had improved; the other reported suicidal ideation at baseline but was lost to follow-up at 10 weeks (purportedly also owing to a travel distance of >500 km) and was contacted by telephone. In post hoc analyses, rates of suicidality (2 weeks: −0.3 [95% CI, −0.5 to 0.1]; 10 weeks: −0.1 [95% CI, −0.5 to 0.3]; *P* = .33), depression (2 weeks: 2.3 [95% CI, 0.3-20.7]; 10 weeks: 2.0 [95% CI, 0.2-19.5]; *P* = .74), or depression severity (2 weeks: −3 [95% CI, −10 to 4]; 10 weeks: −4 [95% CI, −12 to 4]; *P* = .55) did not differ significantly between groups (eTable 5 in Supplement 2).

The most commonly reported moderate adverse event was transient injection site reactions at 2 weeks (degarelix: 22 of 25 [88%]; placebo: 1 of 26 [4%]), and the most commonly reported minor adverse event was hepatobiliary enzyme level elevations, the largest being 3.5 times the upper bound of the normal range (degarelix: 11 of 25 [44%]; placebo: 2 of 26 [8%]). A full description of adverse events and blood sample results is found in eTable 2 and eTable 3 in Supplement 2.

## Discussion

In this phase 2 randomized clinical trial, a single dose (240 mg) of degarelix acetate statistically significantly reduced the dynamic risk factor scores for sexual offense with minimal adverse events among help-seeking men with pedophilic disorder, both in the short (2-week) and in the medium (10-week) terms. The drug was also effective among high-risk participants (Table 3). The rapid onset of degarelix appears to have a crucial advantage compared with earlier medications for paraphilic disorders, which had a 1 to 3 months’ lag in exerting their effects on sexuality.^9^

The self-reports provided an empirical basis for the patient side of shared decision-making and may facilitate patient-centered care for pedophilic disorder.^36^ In weighing the benefits and harms of the drug, we found that the participants self-reported a more positive than negative attitude toward treatment, specifically regarding the effects on sexuality. Thus, participants expressed relief of symptoms for which they sought help, in addition to experiencing the treatment aim of risk reduction. This finding may also be reflected in the reduced risk scores for pedophilic disorder and sexual preoccupation (Table 3). Only 1 participant was lost to follow-up, and 58% of those randomized to receive degarelix wished to continue treatment (Table 4), which indicates to us a potential role for long-term treatment along with psychosocial support in most participants. Ultimately, the treatment decision belongs to the physician, who should take into consideration the risk for abuse, patient preferences, and drug benefits and harms. In view of the participant wishes and effects expressed in the self-reports, we believe degarelix should be considered for help-seeking individuals with pedophilic disorder. The maintained motivation among participants for such potent therapy and the low EQ-VAS ratings reflect the severity and associated distress of the condition. However, a modest effect on quality of life associated with treating the core symptoms of pedophilic disorder indicates the need to better understand the reasons for the struggles experienced by those with this condition.

Future research needs to address the effects and predictable long-term adverse effects of hormone deficiency as well as the sometimes excessive effects on sexuality, as reported by the participants (Table 4; eFigures 3 and 4 in Supplement 2). Incremental add-back therapy of hormones could be considered in this regard.^37^ Given that 2 participants reported severe adverse events of suicidal ideation, vigilance for the risk of exacerbating suicidality in predisposed individuals is warranted. Although depressive symptoms sometimes are manifestations of hypogonadism, a 12-month open-label trial of degarelix in patients with prostate cancer (n = 409) reported depression as an adverse event in only 1 individual.^38^ The high baseline prevalence of depression in the present trial (35%) may indicate an enriched sample of individuals susceptible to depressive deterioration from gonadotropin-releasing hormone antagonist treatment. However, most participants may not be susceptible. In post hoc comparisons of the treatment groups over the study period (eTable 5 in Supplement 2), no differences in change of Montgomery-Åsberg Depression Rating Scale self-rating (MADRS-S) version scores were observed in depression severity, incidence of dysthymia, depression, or suicide risk. Although uncertainty from insufficient power and post hoc analysis remained, most estimates within the CI of the difference in MADRS-S scores at 10 weeks between groups indicated a decrease (10 weeks: −4 [95% CI, −12 to 4]) among participants who were randomized to receive degarelix. When including the MADRS-S scores of all participants, we found that the difference at 10 weeks increased (data not shown).

In addition, future studies need to identify the risk factors that benefit from other kinds of intervention, such as psychotherapy. The impaired self-regulation and low empathy domains in the present trial highlighted the residual risk not amenable to degarelix treatment. Abnormal results in these measures in a clinical setting are usually associated with attention-deficit/hyperactivity disorder or autistic features. We think that if these conditions are adequately addressed, the potential exists to both improve health and reduce the risk for committing child sexual offense.

### Strengths and Limitations

This trial has some strengths. The high inclusion rates, diverse geographic origin of participants, and comorbidity suggest the generalizability of study results to other help-seeking populations. Although few participants reported prior contact offenses, the results are in line with findings of previous unblinded cohorts of convicted offenders, which indicated efficacy of testosterone suppression in reducing sexual symptoms in this population.^12,14^

However, this study also has some limitations. Its results pertain to only men, the risk measure relies mainly on self-reports, and the findings have not yet been validated against actual abuse rates. Therefore, the number of child sexual abuse cases that the reduced risk factor scores have prevented remains to be determined. Furthermore, the prespecified clinically significant reduction of 5 points was not reached, with the exception of participants who were classified as high risk (Table 3).

## Conclusions

Treatment with degarelix appeared to decrease the scores of risk factors for child sexual abuse after 2 weeks of administration for help-seeking men with pedophilic disorder.

## Notes

Supplement 1. Trial Protocol.

Supplement 2. **eAppendix 1.** Methods and Study Details eAppendix 2. Qualitative Content Analysis of Self-Reported Experiences eFigure 1. Change in Composite Risk Score at 2 Weeks from Baseline eFigure 2. Change in Composite Risk Score at 10 Weeks from Baseline eFigure 3. Box-Plot of Sexual Desire Inventory Score by Treatment Group and Timepoint eFigure 4. Box-Plot of Hypersexual Behavior Inventory Score by Treatment Group and Timepoint eTable 1. Additional Baseline Psychiatric Characteristics of Participants eTable 2. Adverse Events eTable 3. Blood Sample Abnormalities eTable 4. Blood Sample Measures eTable 5. Depressive Symptoms and Suicidality eTable 6. Numbers and Proportions (%) of Levels Within EQ-5D Dimensions During the Trial eReferences

Supplement 3. Data Sharing Statement
