# Supplementary material for: Effect of Gonadotropin-Releasing Hormone Antagonist on Risk of Committing Child Sexual Abuse in Men With Pedophilic Disorder: A Randomized Clinical Trial
Source: JAMA Psychiatry. 2020 Apr 29;77(9):1–9. doi: 10.1001/jamapsychiatry.2020.0440 (PMC7191435; doi:10.1001/jamapsychiatry.2020.0440)
Supplement: Supplement 1. — Trial Protocol. [file jamapsychiatry-e200440-s001.pdf]

Protocols

Contents

1. Summary of changes
2. Study protocol
3. Statistical analysis plan

Summary of changes

Protocol changes

The application to the MPA was written in Swedish according to their instructions. It contains the initial protocol (written in Swedish) and statistical analysis plan (written in English), and was approved May19th 2014.

Concurrently, upon initial rejection from the Local Ethical Review Board, the protocol was also adjusted to harmonize with suggested changes from the MPA with the following in the ethical approval of October 22n, 2014.

- In addition to 2 weeks, follow-up at 10 weeks was added.
- Psychopathy Checklist – Revised was replaced by RAADS-14 in the domain of the composite score evaluating empathy.

Before the trial commenced, the final clinical protocol as seen in the CRF was adjusted with the following:

- MINI-antisocial module added as outcome measure to the risk domain empathy.
- Sexual Desire Inventory added as measure to the risk domain sexual preoccupation.
- Conners' Continuous Performance Test and Reading the Mind in the Eyes Revised test were added at 10 weeks to produce identical outcome measures at 2 and 10 weeks.
- Total Sexual Outlet was replaced by SChiMRA as outcome measure to assess the key components pertaining to risk for committing sexual abuse specifically in pedophilic disorder.

The same measures were applied to a healthy control group participating in a case-control study. No adjustments of outcome measures, their interpretation and scoring were done after the trial commenced in March 2016. Because the application for the randomized clinical trial was part of the application for the whole Pedophilia at Risk Investigations for Treatments and Biomarkers (PRIOTAB) project, a large data set including Magnetic Resonance Imaging

(MRI), Functional MRI-data, interviews, blood sampling, and a cohort of healthy controls, it is these other investigations that are stated as the "secondary endpoints" in the applications, and thus motivated the sample size. Although listed among the outcome measures to be used (ethical approval, and final CRF) the secondary endpoints and analyses were not explicitly amended to the approval from the MPA. This is due to the ordinance of the Swedish MPA (ISSN 1101-5225/LVFS 2011:19 Chapter 7 §6), that explicitly deters other than essential changes to be reported to the MPA, and instead refer them to be incorporated to the study documentation, in our case independently monitored by the Karolinska Trial Alliance.

Statistical Analysis Plan amendments.

Statistical analysis of the primary endpoint was performed as stated in the statistical analysis plan in the application to the Swedish Medical Products Agency May 19th 2014 (page 19-20). Before unblinding of treatment allocation, secondary endpoints and methods for analyses of secondary endpoints were amended to the SAP 25th of June, 2019.

PRIOTAB-RCT research plan: A randomized double-blind placebo-controlled study on degarelix effectiveness as an acute treatment drug in pedophilia to reduce the risk of child sexual abuse.

Konfidentiellt

Product

Firmagon

Substance

Degarelix

EudraCT--number

2014---000647---32

Sponsor

Ylva Pernow
Department of Endocrinology, Karolinska University Hospital
Stockholm, Sweden
Sponsor number: 2014:2
Sponsors protocol date: 2014---05---19

Responsible investigator

Christoffer Rahm
Psychiatry Southwest, Karolinska University Hospital Huddinge
Stockholm, Sweden

1. Summary

Protocol identity and aim

EudraCT- number: 2014---000647--32
Title: The Research Project PRIOTAB (Pedophilia at Risk -- Investigations of Treatment and Biomarkers) houses two sub-studies, of which the present project plan describes one, PRIOTAB--RCT. The other, PRIOTAB--CC, is a case control---study that does not involve drugs and therefore is not discussed further here.
The study's objective: The primary purpose of PRIOTAB-RCT is to study degarelix effectiveness as an acute treatment drug for men with pedophilia to reduce the risk of child sexual abuse.

Study drug

Product: Firmagon (degarelix).
Pharmaceutical form: Powder and liquid to injection fluid, liquid 240 mg.
Route of administration: Subcutaneous injection.

Methodology

Study design: Phase 2. Investigator initiated. Double-blind, randomized, placebo-controlled. Two parallel groups.
Dose: At the same time 2 subcutaneous injections degarelix à 120 mg. Evaluation 2 weeks after injection, and after 10 weeks.
Primary hypothesis: Degarelix effective as acute treatment preparations for lowering the risk of child sexual abuse in pedophilia?
Parameter of efficacy: A composite measure of self-rated risk and the four most significant risk factors: abnormal sexual orientation (occurrence of pedophilia), preoccupation of sexual thoughts, lack of self-control and low empathy. Each risk factor is evaluated in terms of (i) self-reported intensity, (ii) neuropsychological testing, as well as potential (iii) presumed biomarker--lab--- and Magnetic resonance imaging--- (MRI---) based investigations.
Safety parameters: 12---lead---EKG, Osteoporosis Self-Assessment Tool for Men (OST), lab (on all visits: fB--glucose, hbA1c and fS--insulin, as well as Na, K, Creatinine, ASAT, ALAT, gamma-GT, ALP) and MADRS-S (self-estimation of depressiveness, includes a question of suicidality). Annual safety and SUSAR reporting, in collaboration with KTA.

Study population

Subjects: Men with pedophilia. (In the second sub-project, PRIOTAB--CC, these men with pedophilia will be compared to a control group that is sex- and age-matched.)
Number: 60 men with pedophilia.

Timetable

First patient included: October 2015.
Last patient included: October 2016.
Last patient finishes: November 2016.

Konfidentiellt

2. Abbreviations

AE	Adverse Event
ASRS	Adult Self Rating Scale
AUDIT/DUDIT	Alcohol / Drug Use Disorders Identification Test
To	Baratt Impulsivity Scale
CASM	Center för andrologi och sexualmedicin
CRF	Case Report Form
CPT	Continuous Performance Test
D2:D4	Finger length ratio between index finger/ring finger left and right
DSM---5	Diagnostic and statistical manual, 5th edition.
DTI	Diffusion Tensor Imaging
EKG	Electrocardiography, 12-lead
EQ---5D	European Quality of Life 5 Dimensions
FDA	Food and Drug Administration
fMRI	Functional MRI
fMRI rs	fMRI resting state
FSH	Follicle stimulating hormone
ICH---GCP	International Conference on Harmonization --- Good Clinical Practice
KTA	Karolinska Trial Alliance
LH	Luteinizing hormone
MINI	Mini International Neuropsychiatric Interview
MRI	Magnetic Resonance Imaging
NRP	Not Real People paradigm
East	Osteoporosis Self-Assessment Tool for Men
PRIOTAB	Pedophilia at Risk --- Investigations of Treatment and Biomarkers
RAADS---14	Ritvo Autism Asperger Diagnostic Scale Screening Tool
RMET	Reading the Mind in the Eyes Test.
SAE	Serious Adverse Event
SchiMRA	Sexual Child Molestation Risk Assessment
SDI/HBI	Sexual Desire Inventory/Hypersexual Behavior Inventory
sMRI	Structural MRI
SSPI	Screening Scale for Pedophilic Interest
SUSAR	Suspected Unexpected Serious Adverse Reactions
TEI	The Edinburgh Inventory
TSO	Total Sexual Outlet

Konfidentiellt

3. Administrative information

Sponsor

Name: Ylva Pernow
Address: Department of Endocrinology, Karolinska University Hospital
Phone: 08-x58580000

Responsible investigator

Name: Christoffer Rahm
Address: M56, Psychiatry Southwest, Karolinska University Hospital Huddinge, 14186 Stockholm
Phone: 0709752291

In 2014, temporary address and phone numbers:
Address: 2/10 Montrose Street, Montmorency, VIC 3094, Australia
Phone: +61 467 027 753

Study nurse

Name: Susanne Jarlvik--Alm
Address: Centrum för andrologi och sexualmedicin (CASM), Endokrinologiska kliniken, KS Huddinge, 141 86 Stockholm Phone: 08- -58 580 000

Study Centre

Name: CASM
Address: Department of Endocrinology, KS Huddinge, 141 86 Stockholm
Phone: 08--58580000 (switchboard)

Laboratory

Name: Karolinska University Laboratory
Adress: L2:03, Karolinska Universitetssjukhuset Solna, 171 76 Stockholm
Phone: 08-x51770000

Monitor

Namn: Karolinska Trial Alliance (KTA)
Address: M62 Karolinska University Hospital Huddinge
Phone: 08-en51774468

Konfidentiellt

4. Background information

Pedophilia

Pedophilia is defined in accordance with DSM-5 (pedophilic disorder, 302.2, F65.4) as sexual attraction patterns toward (prepubertal) children in combination with suffering from it and/or letting it go out over someone else, i.e. committing child sexual abuse or consuming child pornography. Prevalence is estimated to be less than 5% (1). The cause of pedophilia is unclear. It is seen as a chronic condition that has possibly been built already in fetal life and which seems to have genetic elements (2--7). Studies in small forensic psychiatric patient populations have found reduced gray matter volume in ventral striatum, orbitofrontal cortex and cerebellum (8), reduced amygdala on the right side, hypothalamus bilaterally, substantia innominata and "bed nucleus of the stria terminalis " (9), as well as reduced amount of white matter in the upper fronto--occipital fasciculus and right arcuate fasciculus (10). It has also been found different activation patterns in some brain regions in people with pedophilia in visual exposure to images with sexual content. In erotic images of adults, people with pedophilia activate to a lesser extent hypothalamus, insula, occipital cortex, lateral parietal cortex and lateral prefrontal cortex (11), nucleus caudatus (12), gyrus cingulum, gyrus fusiformis, precuneus and temporal-- and occipital cortex (13). By contrast, activation is higher in anterior cingulum cortex, ventromedial prefrontal cortex (12), hippocampus, fusiform gyrus, thalamus (13) and amygdala (14) when exposure to undressed children. About half of child sexual abuse is committed by men with pedophilia (15). The four main risk factors for recidivism in child abuse have been shown to be deviant sexual orientation, lack of self-control, preoccupation in sexual thoughts and low empathy (16). There are several difficulties in the treatment of men with pedophilia. On the one hand, all diagnostics and treatment evaluation are currently based on the person's subjective report, i.e. there are no objective measurement methods for diagnosis, risk evaluation and treatment evaluation in terms of risk of abuse. Furthermore, the treatment methods used are poorly evaluated and tainted with severe side effects. Customary treatment includes psychosocial support, sometimes with psychotherapy, as well as pharmacological treatment. It is very poorly researched what risk factors the different treatments affect. The most widely used pharmacological preparations for high-risk individuals, antiandrogen drugs (cyproterone acetate, leuprolide and triptorelin) all have the downside that they achieve an initial testosterone rise in the first few weeks, with the risk of increased sexual behavior and aggressivity in a sensitive phase of treatment. Time to effect seems to last months to year. There is great need for a drug with more acute effect. Finally, there are knowledge gaps about the neural correlate to pedophilia. The usually reproduced findings are initially mentioned, but a major problem in this field of research is that almost all studies are based on forensic psychiatric clientele and one can assume that they are not representative of the group at large.

Study drug

Degarelix is a GnRH receptor antagonist. It was approved by the FDA in 2008 for the treatment of patients with advanced prostate cancer (17). It hasn't been tested in pedophilia.

Klotz et al (2008) studied its safety and effectiveness in achieving and maintaining testosterone suppression vis-à-vis leuprolide in a comparative, randomized, open--label, parallel—group phase III study over 1 year on a group of 610 patients with prostate cancer (median age 72 years) (18). Three dosing regimens were compared: the starting dose with 240 mg degarelix subcutaneous in a month, followed by maintenance doses of 80 mg or 160 mg monthly, or 7.5 mg leuprolide. Both intention to treat and per protocol populations were analyzed. Primary endpoint, defined as testosterone suppression at less than 0.5 ng/ml blood on all monthly measurements from day 28 to 364, was achieved at 97.2%, 98.3% and 96.4% of patients, in degarelix 240/80 mg, degarelix 240/160 mg and leuprolide groups (intention to treat--population). 3 days after treatment started, testosterone levels were less than 0.5 ng/ml in 96.1% and 95.5% of patients in the degarelix 240/80 mg and 240/160 mg groups, but not in any in the leuprolide--group. However, the hormonal side effects in the three groups were similar in all three groups. The Degarelix groups (subcutaneous injections) had a higher proportion of skin reactions at the plug-in (40%) compared to those in the intramuscular leuprolide--injection group (0%). Other differences between degarelix vs. leuprolide were the incidence of urinary tract infections (3% versus 9% p< 0.01), arthralgia (4% versus 9%, p< 0.05) and chills (4% versus 0%, p< 0.01). Therewere no systemicallergic reactions. The authors concluded that degarelix was no worse than leuprolide in maintaining low testosterone levels over a year. Degarelix lowered testosterone levels markedly faster than leuprolide. The median time for testosterone reinhibitionwas 112 days and the median time to restore testosterone levels was 168 days. The findings in the study of Klotz et al (2008) are supported by studies by Gittelman et al (2008) (19) and Van Poppel et al (2008) (20).

Konfidentiellt

The purpose of the study

Child sexual abuse is a major societal problem that leads to major negative health consequences for those affected and their loved ones. Even on the perpetrator side, there are negative consequences such as social exclusion. The need for primary preventive measures that work is great. Focus has therefore shifted to target the perpetrators. CASM has a unique opportunity to carry out a study targeting non-forensic psychiatric patients with pedophilia and an increased risk of committing abuse. The aim of the study is to examine degarelix effectiveness in reducing the risk of sexual abuse of children in pedophilia. The preparation was chosen because it has the potential to have an acute effect without initial testosterone flare. If it is the case that it helps against certain risk factors but not others, it is interesting to know because then you know what a drug or psychosocial intervention should be directed towards. The study design has been chosen to meet the highest requirements for scientific rigor. The study is investigator-initiated and is free of all forms of association with the pharmaceutical industry.

5. Hypothesis

Primary hypothesis

Is degarelix effective in reducing the risk of child sexual abuse in pedophilia?

Secondary hypotheses

1. What side effects are associated with treatment with degarelix in this patient group?
2. Does the effect of degarelix on of the risk factors correspond to measurable effects also on the supposed biomarkers?

Konfidentiellt

6. Endpoints

Primary endpoint

Primary endpoint is a composite measure 0---15 points, based on estimates scales for various risk factors for sexual abuse. 0--5=low risk. 6-- 10=medium risk. 11--15=high risk. Primary endpoint thus reflects the risk of child abuse, it is not the actual number of child sexual abuse.

Hypothesis for the primary endpoint of PRIOTAB---RCT: The mean change in the composite risk score between baseline and follow up in the degarelix group is equal to that in the placebo group.

The null hypothesis will be tested with a two---sided t---test.

Comment: There are no established measures for the risk of sexual abuse of children in pedophilia (21). The SBU proposes in its 2011 report (22) that endpoint for child sexual abuse should be the number of child sexual abuse. The fact that we refrain from using the proposed endpoint is partly because the measure would be very difficult to get a good idea of-- the under-reporting in research studies presumed to be significant and information taken detour through the judiciary, the number of convictions, probably does not reflect the reality (15). It would also require very large data collections. Furthermore, we find it ethically questionable, with concern for the individual children, to design a study so that it will collect new cases of child sexual abuse. There is therefore a need for a surrogate measure that more focuses on the risk.

We have therefore created this composite measure based on risk factors for sexual abuse. It is based on the results of the only meta-analysis in the field (16). The four risk factors with the highest predictive value of sexual assault that are published in that study (deviant sexual orientation, absorption in sexual thoughts, poor self-control and lack of empathy) are those whose extent may also be presumed to be sensitive to treatment. We also add a fifth item, self-perceived risk. Thus 5 items. Based on the result of estimates of each one, they are scored on a scale of 0--3 points (0=not clinically significant, 1=clinically significant of small scope, 2=clinically significant in medium scope, 3=clinically significant to a high extent). The results are summarized and aggregated points in this composite measure are thus 0--15p. The composite measure is designed by the research team and will be validated in PRIOTAB's first part, PRIOTAB-CC. We believe it has high clinical relevance.

Item 1) Occurrence of pedophilia. Measuring instrument: Screening Scale for Pedophilic Interest (23). Item 2) Sexual preoccupation. Measuring Instrument: Hypersexual Behavior Inventory (24). Item 3) Self-regulating ability. Measuring instrument: Continuous Performance Test (25). Item 4) Empathic ability. Measuring instruments: Reading the mind in the eyes test (26) and Psychopathy check

Konfidentiellt

list -- revised version. Item 5) Current self-rated risk. The Measuring Instrument SChiMRA (Sexual Child Molestation Risk Assessment), developed on CASM.

Konfidentiellt

Secondary endpoints

1. Adverse events, SUSAR, side effects.

2. Effect on supposed biomarkers for risk factors. Hypothesis: The effect of the different treatment arms on the biomarkers of riskfactores differs statistically significantly among themselves.

Risk factor 1. Presence of pedophilia. Measuring instruments: functional MRI/Not Real People Paradigm (27).

Risk factor 2. Busyness with sexual thoughts. Measuring instruments: functional MRI/resting state.

Risk factor 3. Lack of self-regulating ability. Measuring instrument: counting Stroop (28).

Risk factor 4. Low empathy. Measuring instruments: functional MRI/affective faces paradigm.

Sampling in the study

The following samples will be taken at all three visits, to monitor blood glucose levels, kidney-and liver function, as well as to be able to respond to primary and secondary endpoints.

fB--glucose
hbA1c
fS---insulin
Sodium
Potassium
Creatinine
ASAT
Tool
gamma---GT
ALP
S-testosterone
S---SHBG
S--prolactin
S---oxytocin
S---LH,
S---FSH,
S---Ca,
S--estrogen (sensitive),
S--Prolactin

Konfidentiellt

7. Design

Overview design

The study is a investigator-initiated clinical trial, phase 2, Therapeutic exploratory. The design is a double-blind randomized placebo-controlled, parallel groups.

The study design is in accordance with the Helsinki Declaration guidelines. Gold standard for primary prevention of sexual abuse in children with pharmacological treatment of people with pedophilia is lacking, which is why comparison between active substance occurs against placebo (sodium chloride solution).

Randomization is done in advance according to the model "adaptive biased coin--/ urn--design", balanced in blocks, which is the most optimal way in terms of the number of participants. Randomization takes place in advance. Opaque sealed envelopes, as many as the number of subjects in the RCT (N=60), sequentially numbered, contain information about placebo/degarelix located in the same locked cabinet as the trial drug. When a new subject is to be injected, the research nurse takes the top envelope in the pile and provides placebo or degarelix according to instructions in the letter. When baccused interviewers and subjects should be blinded so secret is kept secret for the subject. The letter also has room to document the subject's reserve number. This establishes continuous randomization key. It is defended in the same locked cupboard.

The research participants are completely anonymous to the investigators. This is in order to increase the likelihood that participants are honest in their answers; the shame becomes less towards the interviewer if they are anonymous and they do not have the obligation to notify the SocIal Service Act for the interviewer to take into account in their answers. The anonymisation is carried out in practice by giving the research participant an anonymous medical chart (reserve number) by the research nurse who is the one with the identification key. The investigatorand the researchers are also blinded in terms of what substance the research person has been injected. Only the research nurse has knowledge of this. She has registered this in the same identification key. The key is in paper format and is stored in a binder marked "PRIOTAB" together with the study drugs in a for purpose intended, locked refrigerator on CASM. Even the researchers are blind to what substance they received. The supply of medicines takes place at the end of the visiting period. Evaluation takes placeon weeksand 10 weeks later. The researchers are therefore in the hospital on three occasions. The third investigative moment without an MRI investigation.

Assessments and procedures

The subjects are scheduled to come a total of two full days, for inclusion and for treatment evaluation after two weeks. The third follow-up takes place in meeting with

Konfidentiellt

research nurse. The layout of the first two days looks pretty much the same way:

Visit 1

8:15--8:45 Info, consent (obtained by doctor), registration (research--nurse)
8:45---9:15 Blood sampling, EKG (research-nurse)
9:30---11:00 Medical assessment, self-ratings.
11:00---12:00 Psychological testing
12:00---13:00 Lunch
13:00--14:00 Preparing for MRI investigation
Including instruction and training on fMRI paradigm
14:00--16:00 MRI-investigations, total time in the scanner 50 minutes.
16:00--16:30 Randomization + drug administration + info on continued Contact.

Visit 2

8:15--8:45 Registration (research--nurse)
8:45--9:15 Blood tests (research nurse)
9:30--11:00 Medical assessments, rating scales, AE-reporting.
11:00---12:00 Psychological testing
12:00---13:00 Lunch
13:00--14:00 Preparing for MRI investigation
14:00--16:00 MRI-investigations, total time in scanner 50 minutes

Visit 3

10:00--12:00 Blood tests, estimates, trades, call to enroll as a patient, compensation.

Konfidentiellt

8. Subjects

Inclusion criteria

- Male 18--65 years.
- Diagnosis of pedophilia 302.2 according to DSM-5 (F65.4).
- Signed consent.

Exclusion criteria

- Known severe osteoporosis, or Osteoporosis Self-Assessment Tool for Men (OST) indicates severe osteoporosis.
- The subject may not have extended QT/QTc on the 12-page ECG (>450 ms)we take during screening examination, or have known risk factors for extended QT intervals such as repeated loss of conciousness of unclear etiology, known QT Syndrome in the nearest family, or simultaneous medication with other drugs that may extend the QT range.
- Known reduced liver function, or values outside reference range on the following lab--samples: ASAT, ALAT, ALP, gamma-GT.
- Known severely impaired kidney function, or values outside reference range on the following lab--samples: Sodium, Potassium, Creatinine.
- Severe asthma.
- Known hypersensitivity to the study drug or its excipients.
- Ongoing abuse of alcohol or drugs, measured by AUDIT or DUDIT.
- Counterindications for MRI examination (e.g. implanted metal clip) or other psychologicalas pecter that make it impossible for safe MRI examination (such as claustrophobia).
- Psychological conditions that may compromise patient health or the scientific elements of the study, this is assessed by the investigator (e.g. mental retardation, severe depression, psychosis, hormonal or neurological disorder, etc.).
- Participation in another drug study, within three months before inclusion.

Criteria for suspension

The patient can cancel the study at any time without having to provide reasons. Patients can be taken out of the study prematurely if there are non-tolerable side effects that need to be treated. However, the study follows an intention to treat--ambition which means that data collected until then will be used for analysis and attempts will be made to still get a follow-up even if the person has not completed.

Rejection log

The investigator shall document all patients who have been on screening visits, which have been included and not, and why they have not been included in the study.

Konfidentiellt

9. Treatment

Experimental drug is degarelix (Firmagon) 240 mg (at the same time 2 injections subcutaneous à 120 mg), follow-up after 2 weeks and 10 weeks.

Placebo substance is corresponding volume sodium chloride solution (2 injections subcutaneous), same follow-up time.

Description of the experimental drug

Powder and injection fluid, solution 120 mg. Manufacturer Ferring. The dose of 240 mg that we use in the study is the same as recommended as the starting dose in FASS in prostate cancer and the same as evaluated in the above stated Phase III studies. In addition to active substance, the drug also contains mannitol (facilitates, among other things, the transport of active substance over the blood-brain barrier).

Packing, marking, and handling of the experimental drug

The drugs are purchased through Apoteket, ApoEx, to CASM and stored there in one for the purpose intended locked cabinets (temperature < 25°C) together with identification and randomization keys. The solution is not prepared in advance but in direct connection to administration. If it is waiting, prepared syringes are stored for up to two hours in the same cabinet, waiting will be longer than that, a new syringe will be prepared. We have discussed with Apoteket's clinical trials unit, karolinska university hospital pharmacy hospital pharmacy, about cooperation, but given the short shelf life after preparation and given the transport times between their unit in Solna and our reception in Huddinge, the common assessment is that it is better to have an unblinded research nurse in place instead, at CASM, Huddinge.

How treatment is assigned

The randomization is done in advance according to the model "adaptive biased coin--/ urn-- design" in balanced blocks, which is the most optimal way with respect to the number of participants and practical conditions in general. Opaque sealed envelopes, sequentially numbered and containing information about placebo/degarelix are located in a box in the same locked cabinet. When a new subject is to be injected, the research nurse takes the top envelope in the pile provides placebo or degarelix according to instructions in the letter. Since both interviewers and subjects should be blinded, the info is kept secret for the subject. The letter paper also writes the patient's reserve number. The letter is put back in the same place. This will be our randomization key.

Blinding and code breaking

The research nurse has training in ICH--GCP and great habit of clinical trials. She is unblinded to the will of the preparatory subject. She documents it in the randomization key stored together with the drugs in a locked cabinet in the drug room at CASM. However, the solution is prepared out of sight for the patient. The subject has a plastic card in a walletformat which says that he is involved in a clinical drug trial but not for which preparation ordiagnosis, it also says which

Konfidentiellt

reserve number person has. According to text on the plastic card, it is asked to contact CASM if necessary for code breaking. Research nurse then calls back and gives information to treating doctors about what substance the patient received. This is then recorded in a fail to note logbook. If it is a serious adverse event, the person is taken out of the study (but is included in intention to treat--analysis). Study participants also have a study diary with info about the study and what to do if any health problems related to the study would occur, as well as where they can write down any side effects.

Concomitant medication

Not allowed concurrent medications are hormone preparations that affect testosterone levels, or that are at risk of extending QT-intervallet. The person may not have been involved in any drug study for the past three months, or have used any antiandrogen drugs in the last two years. All other simultaneous treatment is documented in the CRF.

Adherence

The adherence to treatment is assumed to bea complete if the injections could be administered without complications. No drugs should be taken in the home, everything administered on the first day of visits by the research nurse. There is no antidote that the people could take in the home without our knowledge. Vin measures testosterone levels before administration first visit day and on follow-up visits as a marker for antiandrogen effect.

Drug accounting

Research nurse orders the experimental preparation through ApoEx according to the same procedures as for the other drugspurchased to theclinic. She records receiving drugs in the usual way. Administration is documented in the subject's reserve number journal. Possibly leftover study drugs are saved in the clinic's drug storage room if any other patient after thestudy's conclusion would need it, in indication according to FASS (advanced prostate cancer).

Treatment after the end of the study

The subjects are encouraged to enroll as regular patients at CASM after the end of the study, and to present their plastic card with reserve number and information that they have been involved in drug testing. Then the reserve number chart can be linked to the regular charts, still only available within CASM’s TakeCare domain, i.e. it is not visible from other clinics. The patient can also discuss with his doctor about the effects of degarelix/placebo and this will be a basis for decisions on whether the patient should continue with antiandrogen therapy. Since the indication pedophilia is currently lacking in degarelix, one of the andra preparations in the category such as triptorelin (Moapar) or cyproterone acetate (Androcur) are considered.

The effect of degarelix is transient and all patients will regain their regular testosterone levels.

Konfidentiellt

10. Assessment of safety and efficacy

Assessment of clinical effectiveness

The efficacy variables are measured at Karolinska University Hospital Huddinge.

Assessment of clinical safety

Patients are excluded if on the first day of visits turn out to have:

- 12---lead---EKG with QT/QTc---interval> 450 ms.
- Outside reference range on the following samples: Na, K, Creatinine, ASAT, ALAT, gamma-GT, ALP indicating kidney failure or impaired liver function.
- Osteoporosis Self-Assessment Tool for Men (OST) indicating severe osteoporosis
- MADRS-S indicating deep depression, or presence of severe suicidal thoughts

Patients are monitored with respect to glucose intolerance and possible diabetes, with the following samples each visit:

- fB---glucose, hbA1c och fS---insulin

On the first visit, as well as at 2-week----and 10-week follow-up, fB-- glucose, hbA1c and fS--insulin, as well as to Na, K, Creatinine, ASAT, ALAT, gamma-GT, ALP.
All samples are labelled for immediate analysis , and the test results are assessed by doctors on the day of the visit so that medical measures can be taken on the same day.
AE/SAE--reporting takes place in cooperation with KTA.

Analysis response assessment -- efficiency and safety parameters

Efficacy responses are only compiled after the end of the study. However, all patients will be asked to enroll at CASM after participation. Then the current situation and treatment needs can be discussed again. Patients who feel they need urgent medical or psychiatric help during the course of the study are urged to call PrevenTell, or seek psychiatric or medical emergency rooms.

Assessment of clinical safety is carried out continuously. The doctor assesses on the visiting day ECG, OST, lab samples as above and MADRS-S. Unwanted medical events are handled as below. Patients also receive a booklet, a side effects diary, where they can fill in retrospectively what they adverse health effects of treatment they experience, and are encouraged to include it on follow-up.
The annual report, an annual security report to the Swedish Medicines Agency and theEthics Review Committee, will be done in collaboration with KTA.

Konfidentiellt

11. Management of undesired medical events

Definitions

Unwanted medical events (AE) -- Any unwanted medical event or deterioration of current medical conditions (pedophilia), whether it is related to treatment or not.

Unwanted reactions (AR) -- An unwanted reaction is a harmful and accidental medical reaction to a medical product. For an AE to become an AR, the suspected association between the product and the reaction should be at least possible.

Serious unwanted medical events (SAE) -- Any accidental medical event such as:

- results in death
- is life-threatening
- prompts hospitalization or extended hospitalization
- results in residual or temporary handicaps
- results in a congenital injury/birth defect
- is serious for another reason.

Suspected, unforeseen, serious unwanted reactions (SUSAR) -- A reaction/event that is unexpected, serious, is suspected of being caused by the treatment and which is not described before.

Assessment of unwanted medical events

Assessment of severity:

Mild -- The incident does not affect the subject's life.

Moderate -- The event causes deterioration of function with does not affect health. The event causes discomfort and/or inconvenience/obstacles.

Severe -- The event causes deterioration of funktion or incapacity for work or poses a health risk to the person.

Assessment of related links

Probable -- Clinical event, including abnormal laboratory analyses, occurs within a reasonable time after administration of degarelix. Unlikely to the event can be attributed to underlying illness or other drugs.

Possible -- Clinical event, including laboratory analyses, occurs within a reasonable time after administration of degarelix. The incident is unlikely related to degarelix and can be explained by a drug or underlying disease.

Konfidentiellt

Not possible to classify -- The incident cannot be classified because of the absence of information or that the incident is not verified.

Reporting of unwanted medical events

All unwanted medical events are noted in a special AE form in CRF.

Serious unwanted medical events are reported to sponsoron a special SAE form within 24 hours of the fact that we as investigators have become aware of the SAE. Follow-up information describing outcomes and management of the SAE is reported as soon as information is available. The original is inserted into the CRF or trial.

Sponsor is responsible for recording all relevant information about suspicious, unforeseen, serious unwanted reactions and reported to the Swedish Medicines Agency and to the Stockholm Ethics Review Board.

SUSAR, which is deadly or life-threatening, is reported to the Swedish Medicines Agency as soon as possible and no later than 7 days after the incident has become known to sponsor. Relevant follow-up information shall then be submitted within a further 8 days. The rest of SUSAR is reported as soon as possible and no later than within 15 days of being brought to the sponsors' attention.

Follow-up of unwanted medical events

The subject affected by the incident is followed up until the situation is stable and until the drug can be considered to be metabolized. In case of unacceptable events, the subject is taken out of the study and can then obtain testosterone substitution if necessary.

Konfidentiellt

12. Statistics and data handling

Data handling

The research data is handled differently depending on the type of information it is.

Personal data, identification key and randomization key are kept in a binder marked PRIOTAB-study under strict secrecy in specially locked cabinets in the drug room at CASM during the time the study runs and 10 years thereafter. It is then destructed. In the same place, the CRF binder is stored with completed interview templates, test results from neuropsychology and self-rating forms.

Lab data is saved in the reserve number chart. It can be linked to the person's regular medical records if he chooses to become a patient at CASM.

MRI data is stored registered on the reserve number in the X-ray clinic's journal system RIS/PACS linked to TakeCare. That data will be burned off on CDs stored in the CRF binder.

When the study is completed, the randomization key is broken and data is transmitted by hand to encrypted file processed in statistical programs.

Quality control of data is done by responsible investigators.

Statistical analysis

Primary endpoint

This piece of text, about primary endpoint, is authored by our partner in the project in terms of statistics, Professor Matteo Bottai, Core Facility for Biostatistics, Karolinska Institutet:

Primary endpoint is the composite risk score described above, which is measured on a range between 0 and 15 points.

Hypothesis for the primary endpoint of PRIOTAB---RCT: The mean change in the composite risk score between baseline and follow up in the degarelix group is equal to that in the placebo group.

The null hypothesis will be tested with a two---sided t---test. The mean change in the composite factor between baseline and follow---up will be compared between the degarelix and the placebo group.

The power to detect a difference between the degarelix and the placebo groups is calculated as follows. Based on clinical experience we make the following assumptions.

Konfidentiellt

α=0.05 (5% risk of rejecting a correct null hypothesis)
β=0.20 (20% risk of finding support for a false null hypothesis)
$\mu 1 - \mu 2$ (smallest clinically relevant difference in score change) =5
$\sigma = 3$
a = 1,96
b = 0,842
Statistical power=0.80 (1-- β, i.e. 80% chance of rejecting a false null hypothesis) Effect size=u1--u2/ σ=1.7

Statistical power is calculated using this formula:

$N = 2 * (a + b)^2 * \sigma^2 / (\mu 1 - \mu 2)^2 = 2 * (1{,}96+0{,}842)^2 * 3^2 / 5^2 = 6$

Analysis population: The primary endpoint should be analyzed on the population that has taken the study drug and fully responded to the questionnaires so that a full risk assessment could be done on the basis of the composite measure.

To know if the patients should continue, a preliminary analysis of efficacy and safety should be performed when 20 individuals have passed the study. If the results indicate that degarelix has a clinically significant efficacy, there is ethical support to complete the study, so that we also achieve data enough to also be able answering questions related to the secondary endpoints (safety issues and biomarkers for risk assessment, biomarkers for prognosis of medication effect, and biomarkers of medication effect), otherwise PRIOTAB---RCT will be stopped. If the study continues, then a definite analysis will be performed when all of the individuals are collected.

The p---values used at the interim analysis and at the possible final analysis will be 0.0294 [Pocock SJ, Group sequential methods in the design and analysis of clinical trials. Biometrika, 1977, 64 (2): 191–199]. These smaller p---values will preserve the overall level of the test at 0.05.

The blinding will be preserved by not disclosing the treatment group assignment to any of the parties involved, which comprise the patients, the doctors, and the analysts.

Patients that drop out will be included in an intention to treat analysis. Patients with missing values for the risk score at any time point will be excluded from the analysis. The analyses will be performed under the assumption that the missing data generating process is at random [Rubin DB. Inference and missing data. Biometrika, 1976, 63 (3): 581–592]. To assess the plausibility of this assumption, information on the reasons missingness will be elicited from the patients, doctors, or any other source available.

Secondary endpoints

As a secondary endpoint, there is the issue of biomarkers for risk evaluation, treatment outcomes and treatment evaluation.

Statistical analysis for this measure has been done in collaboration with associate professor Andrea Mechelli, Institute of Psychiatry, King's College, London, England, when he

Konfidentiellt

special expertise on the method of analysis to be used -- Unsupervised Machine Learning. It is a method that is adapted to integrate large amounts of clinical data including MRI-based to clinically meaningful dimensions in search of biomarkers, including MRI-based to clinically meaningful dimensions. He has published severalarticles on themethod, see, for example, the 2012 review (29).

Study size

The size of the study is motivated by the secondary objectives and not the primary. Ass Professor Mechelli's recommendation on study size for the secondary objectives is, based on study design in general, N=30 in each treatment arm in PRIOTAB-RCT, (N=60), as well as equal number of controls for comparison in PRIOTAB-- CC.

Konfidentiellt

13. References

1. Seto MC. Pedophilia. Annual review of clinical psychology. 2009;5:391---407.

2. Fagan PJ, Wise TN, Schmidt CW, Jr., Berlin FS. Pedophilia. JAMA : the journal of the American Medical Association. 2002;288(19):2458---65.

3. Bao AM, Swaab DF. Sexual differentiation of the human brain: relation to gender identity, sexual orientation and neuropsychiatric disorders. Frontiers in neuroendocrinology. 2011;32(2):214---26.

4. Swaab DF, Garcia---Falgueras A. Sexual differentiation of the human brain in relation to gender identity and sexual orientation. Functional neurology. 2009;24(1):17---28.

5. Rahman Q, Symeonides DJ. Neurodevelopmental correlates of paraphilic sexual interests in men. Archives of sexual behavior. 2008;37(1):166---72.

6. Jordan K, Fromberger P, Stolpmann G, Muller JL. The role of testosterone in sexuality and paraphilia------a neurobiological approach. Part II: testosterone and paraphilia. The journal of sexual medicine. 2011;8(11):3008---29.

7. Jordan K, Fromberger P, Stolpmann G, Muller JL. The role of testosterone in sexuality and paraphilia------a neurobiological approach. Part I: testosterone and sexuality. The journal of sexual medicine. 2011;8(11):2993---3007.

8. Schiffer B, Peschel T, Paul T, Gizewski E, Forsting M, Leygraf N, et al. Structural brain abnormalities in the frontostriatal system and cerebellum in pedophilia. Journal of psychiatric research. 2007;41(9):753---62.

9. Schiltz K, Witzel J, Northoff G, Zierhut K, Gubka U, Fellmann H, et al. Brain pathology in pedophilic offenders: evidence of volume reduction in the right amygdala and related diencephalic structures. Archives of general psychiatry. 2007;64(6):737---46.

10. Cantor JM, Kabani N, Christensen BK, Zipursky RB, Barbaree HE, Dickey R, et al. Cerebral white matter deficiencies in pedophilic men. Journal of psychiatric research. 2008;42(3):167---83.

11. Walter M, Witzel J, Wiebking C, Gubka U, Rotte M, Schiltz K, et al. Pedophilia is linked to reduced activation in hypothalamus and lateral prefrontal cortex during visual erotic stimulation. Biological psychiatry. 2007;62(6):698---701.

12. Schiffer B, Krueger T, Paul T, de Greiff A, Forsting M, Leygraf N, et al. Brain response to visual sexual stimuli in homosexual pedophiles. Journal of psychiatry & neuroscience : JPN. 2008;33(1):23---33.

13. Schiffer B, Paul T, Gizewski E, Forsting M, Leygraf N, Schedlowski M, et al. Functional brain correlates of heterosexual pedophilia. NeuroImage. 2008;41(1):80---91.

14. Sartorius A, Ruf M, Kief C, Demirakca T, Bailer J, Ende G, et al. Abnormal amygdala activation profile in pedophilia. European archives of psychiatry and clinical neuroscience. 2008;258(5):271---7.

15. Collin---Vezina D, Daigneault I, Hebert M. Lessons learned from child sexual abuse research: prevalence, outcomes, and preventive strategies. Child and adolescent psychiatry and mental health. 2013;7(1):22.

Konfidentiellt

16. Hanson RK, Morton---Bourgon KE. The characteristics of persistent sexual offenders: a meta---analysis of recidivism studies. Journal of consulting and clinical psychology. 2005;73(6):1154---63.

17. FDA. FDA approves drug for patients with advanced prostate cancer. In: administration. USFad, editor. Rockville, MD: FDA;: FDA News.; 2009.

18. Klotz L, Boccon---Gibod L, Shore ND, Andreou C, Persson BE, Cantor P, et al. The efficacy and safety of degarelix: a 12---month, comparative, randomized, open---label, parallel---group phase III study in patients with prostate cancer. BJU international. 2008;102(11):1531---8.

19. Gittelman M, Pommerville PJ, Persson BE, Jensen JK, Olesen TK, Degarelix Study G. A 1---year, open label, randomized phase II dose finding study of degarelix for the treatment of prostate cancer in North America. The Journal of urology. 2008;180(5):1986---92.

20. Van Poppel H, Tombal B, de la Rosette JJ, Persson BE, Jensen JK, Kold Olesen T. Degarelix: a novel gonadotropin---releasing hormone (GnRH) receptor blocker------results from a 1---yr, multicentre, randomised, phase 2 dosage---finding study in the treatment of prostate cancer. European urology. 2008;54(4):805---13.

21. Hanson RK, Morton---Bourgon KE. The accuracy of recidivism risk assessments for sexual offenders: a meta---analysis of 118 prediction studies. Psychological assessment. 2009;21(1):1---21.

22. /SBU LN. Medical and psychological methods for preventing child sexual abuse. In: survey Sbf, editor. Stockholm 2011.

23. Seto MC, Martin L. Lalumière. A brief screening scale to identify pedophilic interests among child molesters. Sexual Abuse: A Journal of Research and Treatment. 2001;13(1):15---25.

24. Reid RC, Garos, S., & Carpenter, B. N. Reliability, validity, and psychometric development of the Hypersexual Behavior Inventory in an outpatient sample of men. Journal of Sexual Addiction & Compulsivity. 2011;18(1):30---51.

25. Conners C.K. SG. Conners' Continuous Performance Test (CPT). In: Kreutzer S.K. DJ, Caplan B., editor. Encyclopedia of Clinical Neuropsychology: Springer New York; 2011. p. 681---3.

26. Fernandez---Abascal EG, Cabello R, Fernandez---Berrocal P, Baron---Cohen S. Test---retest reliability of the 'Reading the Mind in the Eyes' test: a one---year follow---up study. Molecular autism. 2013;4(1):33.

27. Laws D.R. GCLZ. Seeing things differently: The viewing time alternative to penile pletysmography. Legal and Criminological Psychology. 2004;9(2):183---96.

28. Bush G, Whalen PJ, Shin LM, Rauch SL. The counting Stroop: a cognitive interference task. Nature protocols. 2006;1(1):230---3.

29. Orru G, Pettersson---Yeo W, Marquand AF, Sartori G, Mechelli A. Using Support Vector Machine to identify imaging biomarkers of neurological and psychiatric disease: a critical review. Neuroscience and biobehavioral reviews. 2012;36(4):1140---52.

Konfidentiellt

Statistical Analysis Plan

The statistical analysis will proceed as stated in the study protocol.

The primary endpoint is the composite risk score, which is measured on a range between zero and 15 points. The null hypothesis is that the mean change in the composite risk score between baseline and the two-week follow up in the active group is equal to that in the placebo group. We will test this hypothesis with a two-sided t-test at the 0.0294-level. The level is lower than 0.05 to take into account the testing at the interim analysis, as stated in the protocol.

In addition to the primary analysis, we will perform secondary analyses to evaluate differences between the treatment groups with respect to each of the following outcomes separately:

1. Composite risk score (numeric, ranging from 0 to 15)

2. Each of the five sub-scores (numeric ranging from 0 to 3)

3. The composite risk score in a subgroup stated as high risk (10 points or more)

4. European Quality of Life 5-Dimension 3-Level (EQ-5D-3L) index scores (numeric, ranging from 0 to 1)

5. EQ-5D-3L visual analogue scale (numeric, ranging from 0 to 100).

For the secondary analyses, we will include the observations collected at all time-points, baseline, two weeks, and ten weeks. We will analyze the longitudinal data with random effect regression models to allow for potential intra-individual correlation. The regression models will be linear for the numeric outcomes and logit-linked for the binary ones.
All the regression models will include the intercept, an indicator variable for the treatment group, and an indicator variable for the two-week time-point, an indicator variable for the ten-week time-point, and the two interaction (product) terms between the treatment indicator and the time-point indicators. Because patients were randomized to either treatment group, we will include no other covariates. The models will also include a patient-specific random intercept, which we will assume to follow a normal distribution independent of all the covariates.
We will test the difference between the two treatment groups with the Wald's test using the parameter estimates from the random effects regression models.

The number of possible side effects (binary variables) will be summarized by frequency tables by treatment group.

As of Morning 09:14, 25th of June 2019

Matteo Bottai

Matteo Bottai Christoffer Rahm Valdemar Landgren
