# Supplementary material for: Effect of Gonadotropin-Releasing Hormone Antagonist on Risk of Committing Child Sexual Abuse in Men With Pedophilic Disorder: A Randomized Clinical Trial
Source: JAMA Psychiatry. 2020 Apr 29;77(9):1–9. doi: 10.1001/jamapsychiatry.2020.0440 (PMC7191435; doi:10.1001/jamapsychiatry.2020.0440)
Supplement: Supplement 2. — eAppendix 1. Methods and Study Details eAppendix 2. Qualitative Content Analysis of Self-Reported Experiences eFigure 1. Change in Composite Risk Score at 2 Weeks from Baseline eFigure 2. Change in Composite Risk Score at 10 Weeks from Baseline eFigure 3. Box-Plot of Sexual Desire Inventory Score by Treatment Group and Timepoint eFigure 4. Box-Plot of Hypersexual Behavior Inventory Score by Treatment Group and Timepoint eTable 1. Additional Baseline Psychiatric Characteristics of Participants eTable 2. Adverse Events eTable 3. Blood Sample Abnormalities eTable 4. Blood Sample Measures eTable 5. Depressive Symptoms and Suicidality eTable 6. Numbers and Proportions (%) of Levels Within EQ-5D Dimensions During the Trial eReferences [file jamapsychiatry-e200440-s002.pdf]

Supplementary Online Content

Landgren V, Malki K, Bottai M, Arver S, Rahm C. Effect of gonadotropin-releasing hormone antagonist on risk of committing child sexual abuse in men with pedophilic disorder: a randomized clinical trial. *JAMA Psychiatry*. Published online April 29, 2020. doi:10.1001/jamapsychiatry.2020.0440

eAppendix 1. Methods and Study Details

eAppendix 2. Qualitative Content Analysis of Self-Reported Experiences

eFigure 1. Change in Composite Risk Score at 2 Weeks from Baseline

eFigure 2. Change in Composite Risk Score at 10 Weeks from Baseline

eFigure 3. Box-Plot of Sexual Desire Inventory Score by Treatment Group and Timepoint

eFigure 4. Box-Plot of Hypersexual Behavior Inventory Score by Treatment Group and Timepoint

eTable 1. Additional Baseline Psychiatric Characteristics of Participants

eTable 2. Adverse Events

eTable 3. Blood Sample Abnormalities

eTable 4. Blood Sample Measures

eTable 5. Depressive Symptoms and Suicidality

eTable 6. Numbers and Proportions (%) of Levels Within EQ-5D Dimensions During the Trial

eReferences

This supplementary material has been provided by the authors to give readers additional information about their work.

© 2020 Landgren V et al. *JAMA Psychiatry*.

eAppendix 1. Methods and Study Details

List of Investigators

Christoffer Rahm, Principal Investigator

Anna Fernander Hedin, Research Assistant

Pia Jaenssen, Study Nurse

Susanne Jarlvik-Alm, Study Nurse

Stefan Arver, Sponsor

Inclusion and Exclusion Criteria

Eligible patients were male adults age 18-65, referred or self-referred and diagnosed with Pedophilic Disorder according to the Diagnostic and Statistical Manual of Mental Disorders 5. Patients were excluded if they were judged severely mentally unstable by the examining psychiatrist (due to ongoing psychosis, severe depression, mental retardation), could not undergo MRI (due to implants or severe claustrophobia), had participated in a treatment trial within three months of inclusion, interfering treatments with hormonal therapies (such as testosterone receptor blocking medication; antiandrogen therapy), or had conditions stated as contraindications to treatment with degarelix by the Swedish Medical Products Agency, including osteoporosis, QT-c-prolongation >450 milliseconds, liver disease, kidney disease, severe asthma, and ongoing severe substance use disorder.

Common reasons for exclusion in the telephone interview included attraction to pubertal children and not prepubertal children, which is required for a diagnosis of pedophilic disorder.

Randomization Sequence Generation

The Karolinska Trial Alliance provided the independent study nurse with a computer-generated allocation sequence with permuted block randomization.[1]

© 2020 Landgren V et al. *JAMA Psychiatry.*

Description of the trial intervention procedure

At inclusion of a participant, the study nurse opened the corresponding envelope with the card saying whether the participant was to receive study drug or placebo. The study nurse in turn informed the nurse responsible for drug administration. The injection was prepared by the nurse, separate from the participant, by injecting a prefilled syringe of 3 ml sterile water into a vial of powder containing 120 mg degarelix. The vial was swirled until the liquid was clear, and the powder dissolved, and then withdrawn into a syringe. To conceal the trade name, a white sticker was applied to the syringe cylinder, and to further minimize risk of unblinding, the participant was instructed to turn his face away from the nurse before the solution was injected subcutaneously in the abdomen over 30 seconds. This procedure was repeated with a new vial, needle, and syringe for the second 120 mg dose (total dose = 240 mg). The placebo injections consisted of a regular syringe of similar proportions, also with a white sticker on the cylinder, containing 3 ml clear sodium chloride 0.9% injected subcutaneously twice using the same procedure.

Composite Risk Score

Participants were assessed on three self-rated and three expert-assessed measures, all related to sexual preoccupation, self-regulation, cognitive empathy and antisocial traits. The three self-rating scales were: the Sexual Desire Inventory (SDI) measuring sexual interest; the Hypersexual Behavior Inventory (HBI) assessing hypersexuality; and the Ritvo Autism and Asperger Diagnostic Scale – Screening Tool (RAADS-14) mentalizing subscale, a measure of the ability to understand the emotions and behaviors of others. All self-report measures were filled out unsupervised in privacy following the baseline medical examination. The three expert-administered measures were: Conners' Continuous Performance Test – 2nd edition (CCPT-II), testing impulsivity; the Reading the Mind in the Eyes Test Revised Version (RMET), which tests understanding of others' emotions; and the Mini Neuropsychiatric Interview 6.0 Antisocial Personality Disorder symptoms (MINI ASPD symptoms).

© 2020 Landgren V et al. *JAMA Psychiatry.*

Additionally, study participants completed measures addressing sexual deviancy and self-rated risk. We evaluated Pedophilic Disorder symptoms based on DSM-5 criteria and used the Sexual Child Molestation Risk Assessment (SChiMRA) developed by our research group to assess likelihood of sexual offending against children (part A) and past week sexually offensive behavior towards children (part B).

Sexual Desire Inventory-2 (SDI): A self-report measure of frequency and intensity regarding thoughts and feelings about sexual stimuli, both solitary and dyadic (with another person) [2,3]. The adapted Swedish version has omitted item 7 from SDI-2, consisting of 13 items with a score range of 12-104 [4]. A score of <46 is sometimes used in the clinical setting as a cut off when screening for hyposexuality. In a recent systematic review of psychometric properties among measures of sexual desire the SDI was the most frequently used measure [5]. Internal consistency was high (Cronbach's alpha .86 and .96 for the dyadic and solitary dimensions, respectively) [4] and test-retest reliability over a 1-month period was strong (r=.76) [6(p193)]. Regarding convergent validity, SDI total scores correlated significantly with physiological responses to sexual stimuli (r=.18 for the opposite sex and r=.17 for same-sex stimuli). [7].

Hypersexual Behavior Inventory (HBI): A 19-item scale for self-reported symptoms of *hypersexuality*: excessive and uncontrollable sexual fantasies, urges, and behaviors [8]. Hypersexuality is measured through three factors: control, consequences, and coping. The respondent rates negative effects of the sexual behavior, whether the behavior is uncontrollable, and whether sex is used to cope with negative emotions [9]. An example item is "*My sexual behavior controls my life*" and participants indicate their answers on a 5-point Likert scale ranging from 1 (=never) to 5 (=very often) yielding a total score of 19-95. A score ≥ 53 points is considered indicative of hypersexuality [10]. A recent systematic review evaluating psychometric adequacy of the six most researched measurements of hypersexuality

disorder found the HBI to achieve excellent internal consistency (α>.90) and adequate test-retest validity (r>.70) together with good content and construct validity [11].

Ritvo Autism and Asperger Diagnostic Scale – Screening Tool (RAADS-14): A 14-item self-report screening tool for autism spectrum disorder symptoms based on the 80-item scale RAADS-R, developed specifically to assess autistic symptoms in adults [12]. Items cover mentalizing deficits (seven items), social anxiety (four items) and sensory reactivity (three items). An example item is "*It is hard for me to imagine what others expect of me*.". Items are rated on a 4-point Likert scale from 0 (=never true), 1 (=true only when I was younger than 16), 2 (=true only now) and3 (=true now and when I was young). Higher scores therefore reflect more symptoms and symptom persistence. The total score for the full questionnaire is 42, of which half of the points reflect mentalizing deficits [13]. A recent systematic review of nine diagnostic and screening tools for ASD found the RAADS-14 to have satisfactory psychometric properties (with strong evidence for content validity) [14], and excellent internal consistency (α>.90) [13]. Test-retest reliability was not studied.

Conners' Continuous Performance Test (CCPT-II): A performance-based measure covering the domains of inattention, impulsivity, and vigilance [15]. The CCPT-II was administered as part of neuropsychological testing and constitutes a 14-minute computerized test where letters are displayed on a monitor one at a time and the participant must respond to each. The test produces T-scores on 12 aspects of the assignment, comparing the participant to non-clinical and ADHD-norms [16]. These twelve aspects in turn form the three domains inattention (*Omissions, Commissions, Hit RT, Hit RT Std Error, Variability, Detectability (d'), Hit RT ISI Change, Hit RT ISI Change*), impulsivity (*Commissions, Hit RT, Perseverations*) and vigilance (*Hit RT Block Change, Hit SE Block Change*); one of the aspects is not used in any of the domains (*Overall Response Style (ß)).*A study of the test's psychometric properties among psychology students (n=91) found acceptable internal consistency (α ranging from .64

© 2020 Landgren V et al. *JAMA Psychiatry.*

to .96 depending on session) and mostly adequate test-retest reliability (r ranging from .48 to .79 depending on aspect) [15].

Reading the Mind in the Eyes Test, Revised Version (RMET): A tool that attempts to objectively measure an individual's "theory of mind"; that is, the ability to attribute mental states of another person [17]. The revised version [18] consists of 36 pictures depicting an actor's facial expressions, but revealing only the eyes and the area around them. The test subject selects the mental state expressed by the actor in the picture out of four given alternatives. Subjects have access to a dictionary explaining all alternatives and there is no time limit. The possible total score is 36 points, with a higher score reflecting a better ability of emotion attribution. This tool was administered during neuropsychological testing. Two studies of undergraduate students have shown acceptable internal consistency ($\alpha > .60$) and good test-retest reliability (r=.63 and .83) [19,20]. A strong correlation between RMET-scores and self-rated ASD-symptoms (r=.53) has been shown [18] as a measure of convergent validity, although poor concurrent validity has been found between several measures of cognitive empathy [21], indicating that it is a difficult concept to measure.

Mini Neuropsychiatric Interview 6.0 Antisocial Personality Disorder symptoms (MINI ASPD symptoms): These symptoms were assessed with the Mini Neuropsychiatric Interview 6.0: a structured interview covering the DSM-IV diagnostic criteria of the most common, or important, psychiatric disorders [22]. The personality disorder is assessed with twelve items on antisocial behavior and attitudes, coded "yes" or "no". Six items concern the subject at age <15 years and another six ≥15 years. We summarized the number of items with an affirmative response, yielding a score of 0-12 for each participant. The interview was conducted as part of the psychiatrist-led medical examination. The M.I.N.I. 6.0 interview has good test-retest reliability, with r>.75 for a majority of the diagnoses tested. However, agreement with a "gold

© 2020 Landgren V et al. *JAMA Psychiatry.*

standard" semi-structured psychiatric interview, as a measure of validity, was varied [23]. The reliability and validity of the specific ASPD-module has not been tested.

Sexual Child Molestation Risk Assessment (SChiMRA): A generic measure used by our research group comprising self-reported risk of sexual offending (part A), and frequency of sexually offensive behavior towards children (<15 years of age) in the past week (part B). In part A self-reported risk is measured through responding to the question, "*How likely is it that you would do any of the following, if there was an easy way to do it without being caught? Mark an X on the line"* on a visual-analogue scale (0-114 mm) and interpreted as a clinically significant risk if rated at or above 40% (i.e. >45 mm). It covers the three domains of watching CSEM or observing children with sexual intentions; socializing with children with sexual intentions; and direct sexual interaction with children. Part B is self-rated on a 4-point Likert scale, where 0 (=never), 1 (=several days), 2 (=more than half of days), 3 (=almost every day) on the same domains. The total score ranges from 0 to 9.

Additional Psychiatric Characteristics of Participants

Additional baseline characteristics are reported in eTable 1.

Sexual Child Molestation Risk Assessment (SChiMRA)

The instrument is used as instructed under "Detailed description of the Composite Risk Score" above

© 2020 Landgren V et al. *JAMA Psychiatry.*

SChiMRA

Part A

How likely is it that you would do any of the following if there was an easy way to do it without being detected? Mark an X on the line under each question

1) Watch

Watch child sexual abuse material, pictures or films, or discreetly observe children/youths for sexual arousal?

Very likely Not at all

2) Socialize

Socialize/talk to/chat online/call/text/send letters to children/youths for sexual arousal, or in the hopes it may later lead to something more?

Very likely Not at all

3) Interact sexually

Have physical contact with a child/youth for pleasure or sexual enjoyment, or encourage the child/youth into touching you, or stage other types of more direct sexual/sensual situations remotely (for example through webcam)?

Very likely Not at all

©2020 American Medical Association. All rights reserved.

SChiMRA

Part B

Think about the last seven days. How often have you engaged in some of the following:

1) Watched

Watched child sexual abuse material, pictures or films, or discreetly observed children/youths for sexual arousal?

Not at all	A few days	More than half the days	Nearly every day
☐	☐	☐	☐

Comment: ____

2) Socialized

Socialized/talked to/chatted online/texted/sent letters to children/youths for sexual arousal, or in the hopes it may later lead to something more?

Not at all	A few days	More than half the days	Nearly every day
☐	☐	☐	☐

Comment: ____

3) Interacted sexually

Have physical contact with a child/youth for pleasure or sexual enjoyment, or encourage the child/youth into touching you, or stage other types of more direct sexual/sensual situations remotely (for example through webcam)?

Not at all	A few days	More than half the days	Nearly every day
☐	☐	☐	☐

Comment: ____

© 2020 Landgren V et al. *JAMA Psychiatry.*

Results of Interim Analysis

“May 19, 2017

Results of the interim analysis

Anna Hedin [*research assistant*] personally delivered the key to the treatment assignment to me in a closed envelope on May 18, 2017, at 15:30 at IMM [*Institution for Environmental Medicine]* I opened the envelope on May 19, 2017, at 13:15. The key contained 20 patients. I calculated the difference between the baseline and the follow-up measure (difference = follow-up – baseline). The two-sample two-sided t-test for the null hypothesis that mean difference was equal in the two treatment groups showed a p-value of 0.1101. This p-value was above the cutoff of 0.0294 stated in the protocol (page 20). The null hypothesis was not rejected.

Matteo Bottai”

The sponsor decided to continue the study towards the calculated sample size. The decision was taken independently of the principal investigator. There was no evidence of harm from treatment to motivate termination, and secondary endpoints motivated continuation.

Primary and secondary outcomes.

Change in composite risk at 2 and 10 weeks compared to baseline score by treatment group is displayed in eFigure 1 and eFigure 2. Box-plots of the Sexual Desire Inventory (SDI) and Hypersexual Behavior Inventory (HBI) at each timepoint by treatment group is displayed in eFigure 3 and eFigure 4.

Adverse Events and Metabolic Measures

Participants were given a study diary with instructions to note any adverse events in between assessments, and to contact the study nurse by phone if they suspected serious harm from treatment. At follow-up, physical adverse events were registered by the study nurse, using open-ended questions about current health status and the diary. Adverse events were also collected by the assessing psychiatrist, and all events were coded according to the Medical Dictionary for Drug Regulatory Affairs (MedDRA).[24] Reported adverse events are displayed

© 2020 Landgren V et al. *JAMA Psychiatry.*

in eTable 2, and blood sample abnormalities in table S3. Results of blood sample measures are displayed in eTable 4.

Depressive Symptoms and Suicidality

Analyses of depressive symptoms and suicidality by treatment group is reported in eTable 5. No significant differences were found.

The proportion of the binary endpoints (MINI depression and MINI dysthymia) were estimated with logistic random-effects regression models, and the ordinal variables (MINI suicide risk (ranging from 0 to 3) and MADRS-S score (ranging from 0 to 48) among participants with depression) with linear random-effects regression models. Each model included the treatment indicator (binary covariate), indicator variables for the two follow-up visits (binary), and the two interaction terms between the treatment indicator and the two visit indicators (binary). The models also included a subject-specific random intercept, which was assumed to follow a normal distribution. The random intercept was included to take into account the potential dependence in the repeated observations on each subject. We tested for differences in the time trajectories between the treatment groups by testing the composite hypothesis that the interaction terms were jointly equal to zero. All the tests were Wald-type tests. We used the estimates from the models to estimate the mean of the numeric endpoints and the proportions of the binary endpoints. The standard errors used to calculate their confidence intervals were obtained with the Delta method. All the analyses were performed on Stata version 15 (StataCorp, College Station, TX, USA).

© 2020 Landgren V et al. *JAMA Psychiatry.*

Quality of Life

EQ-VAS ratings and EQ5D index distribution between the younger and older half of the total sample are depicted in eFigure 3 and eFigure 4. Post-hoc analyses found no significant differences in VAS or index-scores between age groups at baseline (Mann-Whitney U Test). Numbers and proportions of participants reporting all levels of the EQ-5D health dimensions are found in eTable 6. It has been pointed out that EQ-5D index mainly address physical aspects of health, although the anxiety-dimension is given more weight than other dimensions in the index.[25,26] The VAS-rating of general health may therefore be more suitable for comparisons with patients with other conditions.

© 2020 Landgren V et al. *JAMA Psychiatry.*

eAppendix 2. Qualitative Content Analysis of Self-Reported Experiences

Data Collection

The participants were interviewed during the second visit to the clinic (2 weeks after the injection) and third visit to the clinic (10 weeks after the injection). The interviews were structured and consisted of questions that were formulated by the authors collaboratively. The interviews were held at ANOVA and the participants were interviewed face-to face. Their answers were transcribed in real-time and later translated to English. The following questions were asked:

- Do you think you got placebo or the "real" drug in the injection the last time? Please motivate your answer.

- What positive effects do you experience from the injection?

- What negative effects do you experience from the injection?

One additional question was asked during the visit at 10 weeks:

- Would you like a repeated injection, maintaining the effects for another 10 weeks? Please motivate your answer.

Data Analysis

The answers were analyzed using qualitative descriptive content analysis as described by Sandelowski.[27] Meaning units were marked in the text by the authors and labelled with a code. The codes where later compared in regard to similarity and differences and grouped into sub-categories. [27,28] The sub-categories were abstracted into categories and finally into themes.[28] Participants' treatment allocation (active treatment or placebo) was unknown to the researcher at the time of coding and theme abstraction. Regular meetings were held with the research group to discuss the results. Number of positive and negative effects respectively (binary variables), sorted per sub-category and category, were planned to be summarized by frequency tables by treatment group.

© 2020 Landgren V et al. *JAMA Psychiatry.*

Results of Qualitative Content Analysis
Self-reported Experiences (Degarelix)

What positive/negative effects do you experience from the injection?

Patricpants **(Degarelix group)**	Visit	Meaning units (positive effects)	Code	Subcategory	Category	Theme	Meaning units (negative effects)	Code	Subcategory	Category	Theme
01	2	-					A little tender and sweaty	Tenderness, sweating	Physical symptoms	Negative effects on body	Negative effects of treatment
	3	Thinking less about sex. No unhealthy masturbation. Doesn't get aroused by porn	Decreased sexual interest and behavior	Reduced sex drive	Positive effects on sexuality	Positive effects of treatment	Things I want to be turned on by doesn't turn me on	Doesn't get turned on	Reduced sex drive	Negative effects on sexuality	Negative effects of treatment
02	2	Reduced sex drive	Reduced sex drive	Reduced sex drive	Positive effects on sexuality	Positive effects of treatment	No morning erection, reduced sex drive	No morning erection, reduced sex drive	Reduced sex drive	Negative effects on sexuality	Negative effects of treatment
	3	No sex drive	No sex drive	No sex drive	Positive effects on sexuality	Positive effects of treatment	Sweating, stomach pain	Sweating, stomach pain	Physical symptoms	Negative effects on body	Negative effects of treatment
03	2	Possibly less fixation on sex	Less fixation on sex	Reduced sex drive	Positive effects on sexuality	Positive effects of treatment	Sleep problems, sweating	Sleep problems, sweating	Physical symptoms	Negative effects on body	Negative effects of treatment
	3	Attained a sense of calm. Sexuality has stepped back. Been able to focus on other	Reduced sex drive	Reduced sex drive	Positive effects on sexuality	Positive effects of treatment	Secondary in this context. Reduced sex drive, no erection,	Reduced sex drive, no erection,	Reduced sex drive	Negative effects on sexuality	Negative effects of treatment

© 2020 Landgren V et al. *JAMA Psychiatry.*

	2	things. Was going to separate and live polyamorous. But now I want calm, stabile family life, have kids, and that's me	Establish monogamous adult relationship.	Able to establish adult relationship	Positive effects on relationship		Sweating	Sweating	Physical symptoms	Negative effects on body	
04	2	Less risk to harm children. The sex drive is usually strong, but I feel bad when fantasizing, now I don't have to force these bad fantasies which I need in order to have ejaculation	Reduced sex drive<br>And risk for harming children	Reduces sex drive	Positive effects on sexuality	Positive effects of treatment	Cannot masturbate. Afraid it will lead to ejaculation during the night	Cannot masturbate	Sexual dysfunction	Negative effects on sexuality	Negative effects of treatment
	3	Good that the sexuality disappeared. It was only a source of frustration and consumed a lot of time and mental energy	Sexuality disappeared which was a source of frustration	No sex drive	Positive effects on sexuality	Positive effects of treatment	Sweating	Sweating	Physical symptoms	Negatives effects on body	Negative effects of treatment
05	2	-					In the evenings and 2-3 days after the injection, it hurt by the injection site.	Pain at the injection site	Physical symptoms	Negatives effects on body	Negative effects of treatment

© 2020 Landgren V et al. *JAMA Psychiatry.*

							Hardening at the site of the injection				
	3	Stopped thinking about children. Decreased sex drive. Less frequent masturbation	Less sex drive and interest in children	Reduced sex drive No interest in children	Positive effects on sexuality	Positive effects of treatment	-				
06	2	The stress and pressure and fixation have decreased, which feels good. The constant need to satisfy needs is gone. More important things can take place. Don't need to seek sex for validation. I don't longer play pornographic films in my head then seeing an attractive person. I can see them as humans	Less fixation on sex, no need for constant satisfaction. Sees attractive people as humans	Reduced sex drive	Positive effects on sexuality	Positive effects of treatment	It might be difficult to have ordinary sex If I meet someone	Difficult having normal sex in new relationship	Affected sex life with partner	Relationship problems	Negative effects of treatment
	3	Have become asexual. Feels good. No fantasies about sex or minors during the days. The sex dreams have started to	Asexual. No more sexual fantasies about minors. Doesn't see children and women as sexual objects	No sex drive	Positive effects on sexuality	Positive effects of treatment	Tenderness at the injection site	Tenderness at the injection site	Physical symptoms	Negative effects on body	Negative effects of treatment

© 2020 Landgren V et al. *JAMA Psychiatry.*

		come back. The effect came after 2-3 days. Which was very nice.<br>I think of women and children without sexual tension. They have become humans with feelings. I haven’t been able to think this way my entire life		Different view on children and women	Changed perspective						
07	2	Sex is less important. Relationships are still important	Sex is less important. Relationships are still important	Reduced interest in sex	Positive effects on sexuality	Positive effects of treatment	When I have less sexual activities that can silence my thoughts, I become more preoccupied with thoughts about being lonely. Losing sharpness of thought sometimes	Less sexual activities lead to thought about loneliness.<br>Fewer sharp thoughts	Reduced sexual activities<br>Cognitive symptoms	Negative effects on sexuality<br>Decreased cognitive ability	Negative effects of treatment
	3	Sexual activity has been played down. Decreased desire - feels	Less sexual activity and desire. Not searching for	Reduced sexual interest	Positive effects on sexuality	Positive effects of treatment	Decreased sex drive in general. Had to lie to	Reduced sex drive.	Reduced sex drive	Negative effects on sexuality	Negative effects of treatment

© 2020 Landgren V et al. *JAMA Psychiatry.*

		good. Less active with things I sought help for. Searched 1/3 as many times for illegal porn, not the same need. Less intensity those times I have not been able to resist. The kick is gone regarding sex with younger persons	illegal pornography as much, less intensity				partner about why I couldn't get an erection. Less focus on older (people) also. Sex is an important part of life. Combined with erection medication would be an optimal combination	Cannot get an erection	Affected sex life with partner	Relationship problems	
08	2	Not thinking as much about sex. Doesn't get distracted by sex drive	Reduced sex drive	Reduced sex drive	Positive effects on sexuality	Positive effects of treatment	Pain at injection site. Headache. Also "legitimate" sex drive decreased. Less sexual desire. Erection is still there	Pain, Headache<br><br>Reduced sex drive	Physical symptoms<br><br>Reduced sex drive	Negative effects on body<br><br>Negative effects on sexuality	Negative effects of treatment
	3	No morning erection. Desexualized which feels good. Distance to earlier behavior. The medicine helped me change behavior. Past behavior was	Desexualized. No morning erection. Medicine helped change behavior	No sex drive	Positive effects on sexuality	Positive effects of treatment	Increased shame and self-loathing. Bad for marriage not to be able to have sex. Is horny but can't get an erection	Increased shame and self-loathing<br><br>Cannot get an erection,	Feel shame and self-loathing.<br><br>Sexual dysfunction	Negative emotions<br><br>Negative effect on sexuality	Negative effects of treatment

© 2020 Landgren V et al. *JAMA Psychiatry.*

		irresponsible. I see it as an addiction. A me that is now and one before. Now: children are children. A liberation						bad for marriage	Affected sex life with partner	Relationship problems	
09	2	Not thinking about sex at all. No masturbation	Reduced sex drive	Reduced sex drive	Positive effects on sexuality	Positive effects of treatment	Gassy stomach. Tender swellings on the belly	Abdominal gas, tenderness and swelling	Physical symptoms	Negative effects on body	Negative effects of treatment
	3	Had an unnecessary high sex drive before, now it's less	Reduced sex drive	Reduced sex drive	Positive effects on sexuality	Positive effects of treatment	More gassy stomach	Bloating	Physical symptoms	Negative effects on body	Negative effects of treatment
10	2	A little bit less sex drive	A small reduction in sex drive	Reduced sex drive	Positive effects on sexuality	Positive effects of treatment	-				
	3	The feelings and thoughts about children have disappeared, feels good. Situations that previously caused anxiety are now ok. But there are still exceptions	No sexual feelings and thoughts about children. Less situations-based anxiety	No interest in children<br><br>Less anxiety	Positive effects on sexuality<br><br>Improved mental health	Positive effects of treatment	Have become more sad and tired. Working out doesn't have the same effect	Sadder. More tired.<br><br>Less effect from working out	Sad and tired<br><br>Decreased physical capacity	Mental health issues<br><br>Negative effects on body	Negative effects of treatment
11	2	-					Swelling and tenderness after the injection site, feeling of	Swelling and tenderness. Malaise	Physical symptoms	Negative effects on body	Negative effects of treatment

© 2020 Landgren V et al. *JAMA Psychiatry.*

							inflammation and fever				
	3	-					-				
12	2	Haven't had any sexual thoughts at all. Haven't wanted to browse for pornography. I only cuddle with my girlfriend, no erection. Have had intercourse once, but it was different, not as horny, not the same erection. Sex is not interesting	Sex not interesting. No sex with girlfriend	Reduced sex drive	Positive effects on sexuality	Positive effects of treatment	Pain at injection site for 7 day	Pain at injection site	Physical symptoms	Negative effects on body	Negative effects of treatment
	3	Very positive. What controlled me before (sex drive) is only a memory now. Sense of freedom – feels good. No longer gets stuck in pornography, sex. Feels good not to have sex drive. I want to have children. I am worried about the sexual desire for children and having children	Positive effect. No sex drive. Wants children but worried about the sex drive	Reduced sex drive	Positive effects on sexuality	Positive effects of treatment	Pain for quite some time. Negative for girlfriend, no intercourse	Pain<br><br>No sex with girlfriend	Physical symptoms<br><br>Affected sex life with partner	Negative effects on body<br><br>Relationship problems	Negative effects of treatment

© 2020 Landgren V et al. *JAMA Psychiatry.*

13	2	No sex drive. Interest is gone, harder to become interested in sex. Especially in boring situations. Positive because the energy I have can now be focused on the right things. More even temper. Sex can lead to anxiety. No anxiety over not having done what one intended. Indifference	No sex drive. Not interested in sex. Can focus energy on other things.<br><br>Even temper, less anxiety	No sex drive<br><br>Shifting focus<br><br>Even temper, less anxiety	Positive effects on sexuality<br><br>Changed perspective<br><br>Improved mental health	Positive effects of treatment	Nausea, swelling on the abdomen. Feeling full	Nausea, swelling	Physical symptoms	Negative effects on body	Negative effects of treatment
	3	Decreased sex drive. Controlled sex drive. Shifted focus from thinking about sex to feeling more normal. It saves time. Can deal with other things. I don't procrastinate anymore. E.g. paying back debt. Grocery shopping in time. Everyday things. I feel much better mentally. A slightly different	Decreased and controlled sex drive.<br><br>Can focus on other things now.	Reduced sex drive.<br><br>Shifting focus	Positive effects on sexuality<br><br>Changed perspective	Positive effects of treatment	-				

© 2020 Landgren V et al. *JAMA Psychiatry.*

		person. Change to the positive. More harmonious	Feeling better mentally and more harmonious	More harmonious	Improved mental health						
14	2	Less anxiety. Fewer sexual thoughts	Less anxiety. Fewer sexual thoughts	Reduced sexual thoughts<br><br>Less anxiety	Positive effects on sexuality<br><br>Improved mental health	Positive effects of treatment	Pain related to the injection. Feeling of a cold 1-2 days after the injection	Pain. Malaise	Physical symptoms	Negative effects on body	Negative effects of treatment
	3	Can focus more on the 12-step program. Fewer sexual thoughts	More focus on other things<br><br>Fewer sexual thoughts	Shifting focus<br><br>Reduced sexual thoughts	Changed perspective<br><br>Positive effects on sexuality	Positive effects of treatment	Sweating. Flushing. Pain at injection site	Sweating. Hot flush. Pain at injection site	Physical symptoms	Negative effects on body	Negative effects of treatment
15	2	-					Pain at the site of injection	Pain	Physical symptoms	Negative effects on body	Negative effects of treatment
	3	-									
16	2	-					Pain at the site of injection	Pain at the site of injection	Physical symptoms	Negative effect son body	Negative effects of treatment
	3	Decreased sex drive. You focus on the right things, i.e. don’t seek sex from the wrong person, i.e. minors	Decreased sex drive	Reduced sex drive	Positive effects on sexuality	Positive effects of treatment	Less sex drive - boring in the eyes of others	Decreased sex drive and boring	Reduced sex drive	Negative effects on sexuality	Negative effects of treatment

© 2020 Landgren V et al. *JAMA Psychiatry.*

17	2	No suicidal thoughts any more, not depressed	No suicidal thoughts, not depressed	Reduced psychiatric symptoms	Improved mental health	Positive effects of treatment	Abdominal swelling. Weakness in the body for the first 2 days. About 40% weaker. Affected work (manual laborer)	Abdominal swelling. Weakness	Physical symptoms	Negative effects on body	Negative effects of treatment
	3	Browsing pornographic sites has decreased. Less sexuality, doesn't want it	Less porn browsing. Less sexually	Reduced sex drive	Positive effects on sexuality	Positive effects of treatment	The pain afterwards. Physical capacity decreased. Bad for work, manual work. I should have had time off (from work).	Pain<br><br>Decreased physical capacity<br><br>Affected work, should have taken time off	Physical symptoms<br><br>Decreased physical capacity<br><br>Affected work	Negative effects on body<br><br>Negative effects on work	Negative effects of treatment
18	2	Less preoccupation with sexual thoughts, noticed after two days. Less bothered by distracting sounds (e.g. hearing children on the playground)	Less preoccupation with sexual thoughts. Less disturbed by distracting sounds	Reduced sexual thoughts	Positive effects on sexuality	Positive effects of treatment	A little more tired	Fatigue	Physical symptoms	Negative effects on body	Negative effects of treatment
	3	Reduced sex drive, easier to concentrate,	Reduced sex drive.	Reduced sex drive	Positive effects on sexuality	Positive effects of treatment	-				

© 2020 Landgren V et al. *JAMA Psychiatry.*

		easier to handle difficult situations	Easier to concentrate and handle difficult situations	Easier to concentrate	Improved cognitive ability						
19	2	Reduced sex drive	Reduced sex drive	Reduced sex drive	Positive effects on sexuality	Positive effects of treatment	Abdominal pain the first few days. “But pain is just another form of pleasure”	Abdominal pain	Physical symptoms	Negative effects on body	Negative effects of treatment
	3	Sees other pleasures in life. More at peace. Can talk to friends without thinking about wanting to go home and browse for pornography. Enjoys the everyday life. Sees the little joys. Can renovate the moped without feeling like wasting time instead of surfing for pornography	More at peace and not focused on porn	No interest in porn	Positive effects on sexuality	Positive effects of treatment	Feeling very warm. Increased body temperature	Fever	Physical symptoms	Negative effects on body	Negative effects of treatment
20	2	Fewer thoughts about sex	Fewer thoughts about sex	Reduced sex drive	Positive effects on sexuality	Positive effects of treatment	Stomach pain	Stomach pain	Physical symptoms	Negative effects on body	Negative effects of treatment
	3	-					-				
21	2	-					-				

© 2020 Landgren V et al. *JAMA Psychiatry.*

	3	Fewer thoughts and feeling connected to children changing clothes. Those that come can more easily be pushed back. This is due to the problem. Feels better because I don't really want those thoughts. I know you're not supposed to think like that	Less thoughts about children. Can push back thoughts	Reduced sexual thoughts of children	Positive effects on sexuality	Positive effects of treatment	-				
22	2	-					Stomach pain the first few days, probably because of the needle	Stomach pain	Physical symptoms	Negative effects on body	Negative effects of treatment
	3	Can focus more on what is important: relationships, emotional response	Focus on more important stuff	Shifting focus	Changed perspective	Positive effects of treatment	Stomach pain. If the opportunity for sex arises, won't be as interested	Stomach pain. Less interested in sex	Physical symptoms<br><br>Reduces sexual interest	Negative effects on body<br><br>Negative effect on sexuality	Negative effects of treatment
23	2	-					Stomach pain. Fatigue	Stomach pain. Fatigue	Physical symptoms	Negative effects on body	Negative effects of treatment
	3	No attraction to minors. Not looked at pictures or watched pornos of	No sexual interest to children	Reduced sexual interest	Positive effects on sexuality	Positive effects of treatment	Gets painful erections. Gets orgasm but no ejaculation	Painful erection. No ejaculation	Sexual dysfunction	Negative effects on sexuality	Negative effects of treatment

© 2020 Landgren V et al. *JAMA Psychiatry.*

		children, it's different to how it's used to be									
24	2	-					Large swelling on abdomen, hurt when sitting and standing. Extremely loose ejaculate, watery	Swelling and pain on abdomen<br><br>Watery ejaculation	Physical symptoms<br><br>Sexual dysfunction	Negative effects on body<br><br>Negative effects on sexuality	Negative effects of treatment
	3	-					Very watery ejaculation. Worried about having gotten the real drug and that I am immune to the treatment regarding the effects on sexuality	Watery ejaculation. Worrying about being immune to the drug	Sexual dysfunction	Negative effects on sexuality	Negative effects of treatment
25	2	-					Erectile dysfunction	Erectile dysfunction	Sexual dysfunction	Negative effects on sexuality	Negative effects of treatment
	3	Can better handle the sexual thoughts	Can better handle the sexual thoughts	Control over sexual thoughts	Improved self-control	Positive effects of treatment	Hot flushes, erectile dysfunction	Hot flushes.<br><br>Erectile dysfunction	Physical symptoms<br><br>Sexual dysfunction	Negative effects on body<br><br>Negative effects on sexuality	Negative effects of treatment

© 2020 Landgren V et al. *JAMA Psychiatry.*

Self-reported Experiences (Placebo)

What positive/negative effects do you experience from the injection?

Participants **(Placebo group)**	Visit	Meaning units (positive effects)	Code	Subcategory	Category	Theme	Meaning units (negative effects)	Code	Subcategory	Category	Theme
26	2	-					Difficulty concentrating	Difficulty concentrating	Cognitive symptoms	Decreased cognitive ability	Negative effects of treatment
	3	-					-				
27	2	-					-				
	3	-					-				
28	2	-					Fever in the beginning, warm/cold	Fever	Physical symptoms	Negative effects on body	Negative effects of treatment
	3	Lost sex drive. That's good, because it felt unnecessary to have	Loss of sex drive	Loss of sex drive	Positive effects on sexuality	Positive effects of treatment	Fever in the beginning	Fever	Physical symptoms	Negative effects on body	Negative effects of treatment
29	2	-					More tired<br><br>Hornier. Wants to masturbate more	More tired<br><br>Hornier. Masturbating more	Physical symptoms<br><br>Increased sex drive	Negative effects on body<br><br>Negative effects on sexuality	Negative effects of treatment
	3	-					-				
30	2	-					-				
	3	-					-				

© 2020 Landgren V et al. *JAMA Psychiatry.*

31	2	-					Headache	Headache	Physical symptoms	Negative effects on body	Negative effects of treatment
	3	Increased energy. Better appetite, eating more, sleeping better	Increased energy, better appetite and sleep	Increased energy and better sleep	Improved physical health	Positive effects of treatment	Headache	Headache	Physical symptoms	Negative effects on body	Negative effects of treatment
32	2	-					-				
	3	At setbacks and boredom, I no longer search for child pornography. Positive as this is self-harming behavior	Does not watching child pornography at setbacks. See it as self-harming behavior	No interest in child pornography	Positive effects on sexuality	Positive effects of treatment	Less desire for sex with partner	Less desire for sex with partner	Reduced sex drive	Negative effects on sexuality	Negative effects of treatment
33	2	-					Tenderness at the injection site	Tenderness at injection site	Physical symptoms	Negative effects on body	Negative effects of treatment
	3	Possibly a little less desire	A small reduction in sex drive	Reduced sex drive	Positive effects on sexuality	Positive effects of treatment	Swelling at the injection site. Discomfort in testicles	Swelling. Testicle discomfort	Physical symptoms	Negative effects on body	Negative effects of treatment

© 2020 Landgren V et al. *JAMA Psychiatry.*

34	2	Easier to refrain from watching child porn	Easier to refrain from watching child porn	Refrain from child porn	Improved self-control	Positive effects of treatment	-				
	3	Easier to abstain from going online and searching for pornography	Easier to abstain from going online and searching for pornography	Abstain from child porn	Improved self-control	Positive effects of treatment	-				
35	2	-					-				
	3	I don't have the sexual fantasies I had before – it is very positive	Decreased sexual fantasies	Reduced sex drive	Positive effects on sexuality	Positive effects of treatment	I don't feel any sexual desire for my wife either	Reduced sexual attraction to wife	Affected sex life with partner	Relationship problems	Negative effects of treatment
36	2	-					-				
	3	-					-				
37	2	-	-		-						
	3	-					-				
38	2	-					-				

© 2020 Landgren V et al. *JAMA Psychiatry.*

	3	-					-				
39	2	-					-				
	3	Decreased sexual compulsiveness regarding children. I Used to masturbate to thoughts about children 4-5 times per week, now twice per week	Decreased sexual compulsiveness regarding children. Less masturbation	Reduced sex drive	Positive effects on sexuality	Positive effects of treatment	Feeling lower for a period in the middle of treatment	Feeling low	Feeling low	Mental health issues	Negative effects of treatment
40	2	-					A little tired	Tired	Physical symptoms	Negative effects on body	Negative effects of treatment
	3	-					-				
41	2	-					Possibly back pain, but it is probably related to working in front of the computer	Back pain	Physical symptoms	Negative effects on body	Negative effects of treatment
	3	-					-				
42	2	-					Feels genitals have become smaller	Smaller genitals	Physical symptoms	Negative effects on body	Negative effects of treatment
	3	Although the emotional attraction to boys remains, the sexual interest has decreased	Less sexual interest. Emotional attrac	Reduced sexual interest	Positive effects on sexuality	Positive effects of treatment	I am married and expected to have intimacy with my wife, but decreased sex drive counteracts that. I would like to feel sexual desire	Decreased sex drive	Decreased sex drive	Negative effects on sexuality	Negative effects of treatment

© 2020 Landgren V et al. *JAMA Psychiatry.*

			tion to boys remains					Affected sex life with wife	Affected sex life with wife	Relationship problems	
43	2	-					-				
	3	-					-				
44	2	Reduced sex drive. Feels good, doesn't interrupt my thoughts. Easier to focus on other things	Reduced sex drive, easier to focus on other things	Reduced sex drive<br><br>Focus on other things	Positive effects on sexuality<br><br>Changed perspective	Positive effects of treatment	Possibly more mood swings	Mood swings	Mood swings	Mental health issues	Negative effects of treatment
	3	-					-				
45	2	No longer need to watch pornography. Feels good not to. Thereby the risk is lower to cross over to illegal pornographic material. Watching it is consuming. Leads to internal fragmentation. Worth gold!	Less interest in watching pornography	Reduced sexual interest	Positive effects on sexuality	Positive effects of treatment	-				
	3	Reduced sex drive. Minimizes the risk of watching illegal	Reduced sex drive,	Reduced sex drive	Positive effects on sexuality	Positive effects of treatment	-				

© 2020 Landgren V et al. *JAMA Psychiatry.*

		material. Good that it's a long-acting treatment	minimizing risk of watching illegal material								
46	2	A noticeable decrease in impulses, the urge for sex is gone, masturbation works a lot different – mostly to test, no pleasure or any other sensations, no compulsion	Decreases impulse and sexual urge, less pleasure of masturbation	Reduced sex drive	Positive effects on sexuality	Positive effects of treatment	-				
	3	Incredibly good! An amazing calm. A feeling of not having to masturbate. Could start focusing on other things. Sex is energy consuming	Don't have to masturbate. Focus on other things	Reduced sex drive<br><br>Focus on other things	Positive effects on sexuality<br><br>Changed perspective	Positive effects of treatment	Restlessness, sweating, peeing more often	Restlessness, sweating, peeing more	Physical symptoms	Negative effects on body	Negative effects of treatment
47	2	-					Stomach has "crashed". Loose stool. Irregular	Gastro-intestinal issues. Tired	Physical symptoms	Negative effects on body	Negative effects of treatment

© 2020 Landgren V et al. *JAMA Psychiatry.*

							bowel movements. More tired but was tired before too				
	3	Effect on sex drive is good. Doesn't get turned on sexually. Less need for wanting sex. Can look at a person and think: attractive. But without WANTING the person, sexually. Good because it's a risk of committing abuse that now has disappeared. Can see without wanting to touch	Redu ced sex drive	Reduced sex drive	Positive effects on sexuality	Positive effects of treatment	No negative effects. Shaky, anxious	Shaky and anxious	Anxious	Mental health issues	Negative effects of treatment
48	2	-					-				
	3	-					-				
49	2	Sexual preoccupation has decreased. The sexualization of everything stopped. Sexuality has become more adequate. It doesn't stain everything like it used to. For example, in an ordinary conversation;	Redu ced sex drive. But starti ng to come back	Reduced sex drive	Positive effects on sexuality	Positive effects of treatment	Loss of energy and less able to deal with things. Sex drive decreased too much in the beginning. Feeling more low than usual.	Less energy. Feeling low.<br><br>Reduced sex drive	Lethargic, feeling low<br><br>Reduced sex drive	Mental health issues<br><br>Negative effects on sexuality	Negative effects of treatment

© 2020 Landgren V et al. *JAMA Psychiatry.*

		before it would become too much. It was hard. Now I don't say inappropriate things. Fewer sexual associations. It was reduced at first. Now it's starting to come back though. But not as much as when it was at its highest. Calmer than usual								
	3	Easier to take charge in sexual situation and stop. Sex became less important. A general reduction in sex drive. Less sexual impulses	Reduced sex drive, less sexual impulses	Reduced sex drive	Positive effects on sexuality	Positive effects of treatment	-			
50	2	More confident. More mature behavior - playing less on the computer, spending more time with parents, watching drama shows on TV and news.	More confident and mature behavior. Less computer game	More confident and mature behavior<br><br>More family time	Improved mental health<br><br>Changed perspective	Positive effects of treatment	Weight gain, more lethargic	Weight gain, more lethargic	Physical symptoms	Negative effects on body	Negative effects of treatment

© 2020 Landgren V et al. *JAMA Psychiatry.*

			s, more famil y time								
	3	More calm. Increased self-esteem	More calm. Incre ased self-estee m	Increased calmness and self-esteem	Improved mental health	Positive effects of treatment	-				
51	2	-					Rash on the face and arms. Heat sensitivity	Skin rash	Physical symptoms	Negative effects on body	Negative effects of treatment
	3	-					Temporary skin rash. Heat rash	Skin rash	Physical symptoms	Negative effects on body	Negative effects of treatment

© 2020 Landgren V et al. *JAMA Psychiatry*.

Willingness to Continue Treatment (Degarelix)

Would you like a repeated injection maintaining the effects for another 10 weeks? Please motivate your answer

Participants **Degarelix group**	Take inj again? (Yes/No)	Meaning unit	Condensed meaning unit	Code	Subcategory	Category	Theme
01	N	No. I want to engage in sexual activities again, but not children. This was a good time-out to break bad habits. Would take it again if I need it.	Want to engage in sexual activities again, but not children.	Lack of sexual activities	Lack of sexual activities	Negative effects on sexuality	Reasons for discontinuing treatment
			Good time-out to break bad habits. Take again if needed	Take injection if needed again	Medication when needed	Positive attitude	Attitudes to treatment
02	N	No. I don't feel the need. The medication helped me change the behavior. But don't need it anymore	Don't feel the need anymore. The medication helped to change the behavior	Changed behavior	Broken behavior pattern	Achieved effect	Reasons for discontinuing treatment
03	N	No, due to loss of erection	Loss of erection	Erectile dysfunction	Erectile dysfunction	Negatiev effects on sexuality	Reasons for discontinuing treatment
04	N	No	-				

© 2020 Landgren V et al. *JAMA Psychiatry.*

05	N	No, I want to be careful with which medical substances I take. But will take it again if needed	Want to be careful with intake of medicine<br><br>Will take it again if needed	Medicine intake<br><br>Take injection if needed	Caution taking medication<br><br>Medicate when needed	Cautiousness<br><br>Positive attitude	Reasons for discontinuing treatment<br><br>Attitudes to treatment
06	N	No, because of no effect	No effect	No effect	No effect	No effect	Reasons for discontinuing treatment
07	N	No, the pain was unpleasant. Don't need it anymore. Would recommend other in the same situation to take it again	Unpleasant pain<br><br>No longer needing it<br><br>Would recommend to others in the same situation	Pain<br><br>No further need<br><br>Would recommend treatment to others	Physical symptoms<br><br>No further need<br><br>Recommendation	Negative effects on body<br><br>Achieved effect<br><br>Positive attitude	Reasons for discontinuing treatment<br><br>Attitudes to treatment
08	-	-	-	-	-	-	

© 2020 Landgren V et al. *JAMA Psychiatry.*

09	N	No. Swelling at the injection site was to problematic	Swelling at injection site	Swelling	Physical symptoms	Negative effects on body	Reasons for discontinuing treatment
10	N	No. Adverse effects	-				

11	Y	Yes, why not. Would like to have it as maintenance treatment. Doesn't take away the hate toward little girls, could still hit them.	Would like to have it as maintenance treatment, doesn't affect the hate toward girls.	Continue treatment	Maintenance treatment	Positive attitude	Attitudes to treatment
12	Y	Yes. Feels like there is hope about life	Feeling hopeful about life	Hopefullness	Feel hopeful	Positive emotions	Reasons for continuing treatment
13	Y	Yes. I don't want the sex drive back, buy a new computer, internet, start abusing. Doesn't trust myself.	Doesn't want the sex drive back. Doesn't trust myself, no self-control	No sex drive	No sex drive	Positive effects on sexuality	Reasons for continuing treatment

© 2020 Landgren V et al. *JAMA Psychiatry.*

		Cannot control myself					
14	Y	Yes. Want to try a period without medicine. But after that, yes	Wants to try a medicine- free period first	Continue treatment	Medicine-free period	Positive attitude	Attitudes to treatment
15	Y	Yes. A relief. But perhaps a weaker dose. Would like to have a stronger erection	A relief. Weaker dose due to erection problem	A relief<br><br>Changed dosage	Relief<br><br>Sexual dysfunction	Positive emotions<br><br>Overall positive effects	Reasons for continuing treatment
16	Y	Yes	-				
17	Y	Yes, but want to feel more alert	Yes, wants to feel more alert	Feeling tired	Continue medication despite of fatigue	Overall positive effects	Reasons for continuing treatment
18	Y	Yes	-				
19	Y	Yes, but would like to wait. Would take it again if there is a crisis with increased sexuality again	Yes, but would wait. Would take again if increased sexuality	Continue treatment when needed	Medicate when needed	Positive attitude	Attitudes to treatment
20	Y	Yes	-				
21	Y	Yes	-				
22	Y	Yes. Hoping that I eventually would become clear headed and manage without	Hope to manage without medicine, but I need it a while longer	Continue treatment	In need of medication	Necessary	Reasons for continuing treatment

© 2020 Landgren V et al. *JAMA Psychiatry.*

		medicine. But I need it a little while longer					
23	Y	Yes (but fear of needles	-				
24	Y	Yes	-				
25	Y	The positive outweigh the negative	The positive outweigh the negative	The positive outweigh the negative	The positive outweighs the negative	Overall positive effects	Reasons for continuing treatment

Willingness to Continue Treatment (Placebo)

Would you like a repeated injection maintaining the effects for another 10 weeks? Please motivate your answer

Participants (**Placebo group)**	Take injection again? (Yes/No)	Meaning unit	Condensed meaning unit	Code	Subcategory	Category	Theme
26	N	No. I want the real medicine	Wants the real medicine	No effect	No effect	No effect	Reasons for discontinuing the treatment
27	N	No. There is no point	-				
28	N	No to injection, yes to pills.	Painful injection	Pain	Physical symptoms	Negative effect on body	Reasons for discontinuing the treatment

© 2020 Landgren V et al. *JAMA Psychiatry.*

		Because the injections hurts					
29	N	No, nothing happened!	No effect	No effect	No effect	No effect	Reasons for discontinuing the treatment
30	N	No, because there was no effect	No effect	No effect	No effect	No effect	Reasons for discontinuing the treatment
31	N	No	-				
32	N	No, want something that works, not what I got this time	Wants something that works	No effect	No effect	No effect	Reasons for discontinuing the treatment
33	N	No	-				
34	N	No, it had no effect	No effect	No effect	No effect	No effect	Reasons for discontinuing the treatment
35	N	No	-				
36	N	No effect	No effect	No effect	No effect	No effect	Reasons for discontinuing the treatment
37	N	No	-				
38	N	No	-				
39	N	No. Wants the real injection! No if the same, yes if the real one.	Wants the real medicine	No effect	No effect	No effect	Reasons for discontinuing the treatment
40	N	No	-				

© 2020 Landgren V et al. *JAMA Psychiatry.*

41	N	No. It had no effect	No effect	No effect	No effect	No effect	Reasons for discontinuing the treatment
42	N	No. It had no effect. Seems pointless	No effect	No effect	No effect	No effect	Reasons for discontinuing the treatment
43	Y	Yes. Positive if it becomes a criminal case	Positive if it becomes a criminal case	Criminal case	Affect criminal case	Legal matter	Reasons for continuing treatment
44	Y	Yes. It has been worth it	-				
45	Y	Yes	-				
46	Y	Yes. A couple more (injections) to get into new patterns of thoughts	More injection to change thought pattern	Continue treatment	To change behavior	To achieve effect	Reasons for continuing treatment
47	Y	Yes	-				
48	Y	Yes, but it also feels like it doesn't matter	-				
49	Y	Yes, but would first like to try a period without	Try a medicine-free period first	Continue treatment	Medicine-free period	Positive attitude	Attitudes to treatment
50	Y	Yes. Solves a lot of problems in life, with relationships	Solves problems in life, with relationships	Problem-solution	Solving relationship problems	Positive effects on relationship	Reasons for continuing treatment
51	Y	Yes	-				

© 2020 Landgren V et al. *JAMA Psychiatry.*

Patient Beliefs About Treatment Allocation

"Do you think you got placebo or the "real" drug in the injection the last time? And motivate why"

At 2 and 10 weeks respectively, 16 out of 25 (64%) and 22 out of 24 (92%) in the group assigned degarelix and six (23%) and eleven (42%) out of 26 in the group assigned placebo believed they had received active treatment.

© 2020 Landgren V et al. *JAMA Psychiatry.*

eFigure 1. Change in Composite Risk Score at 2 Weeks from Baseline

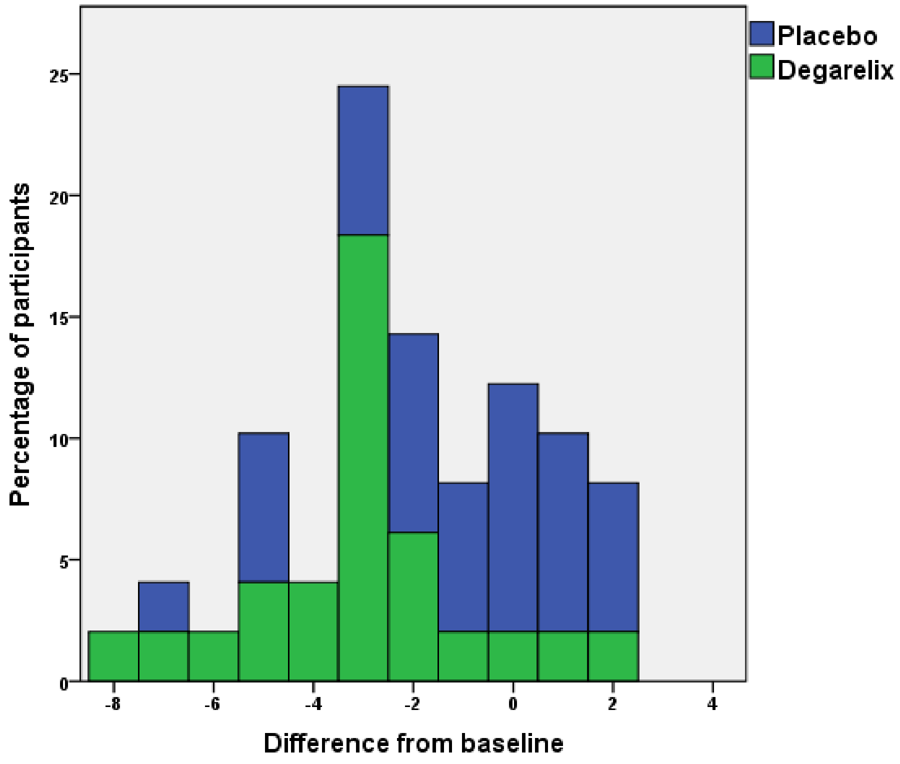

© 2020 Landgren V et al. *JAMA Psychiatry.*

eFigure 2. Change in Composite Risk Score at 10 Weeks from Baseline

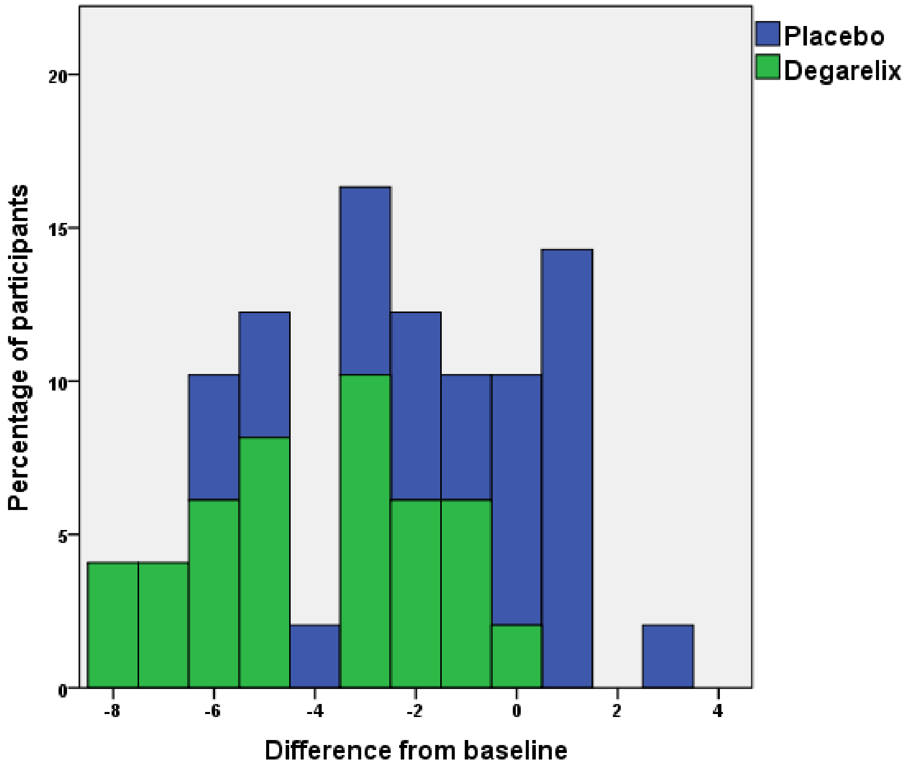

© 2020 Landgren V et al. *JAMA Psychiatry.*

eFigure 3. Box-Plot of Sexual Desire Inventory Score by Treatment Group and Timepoint

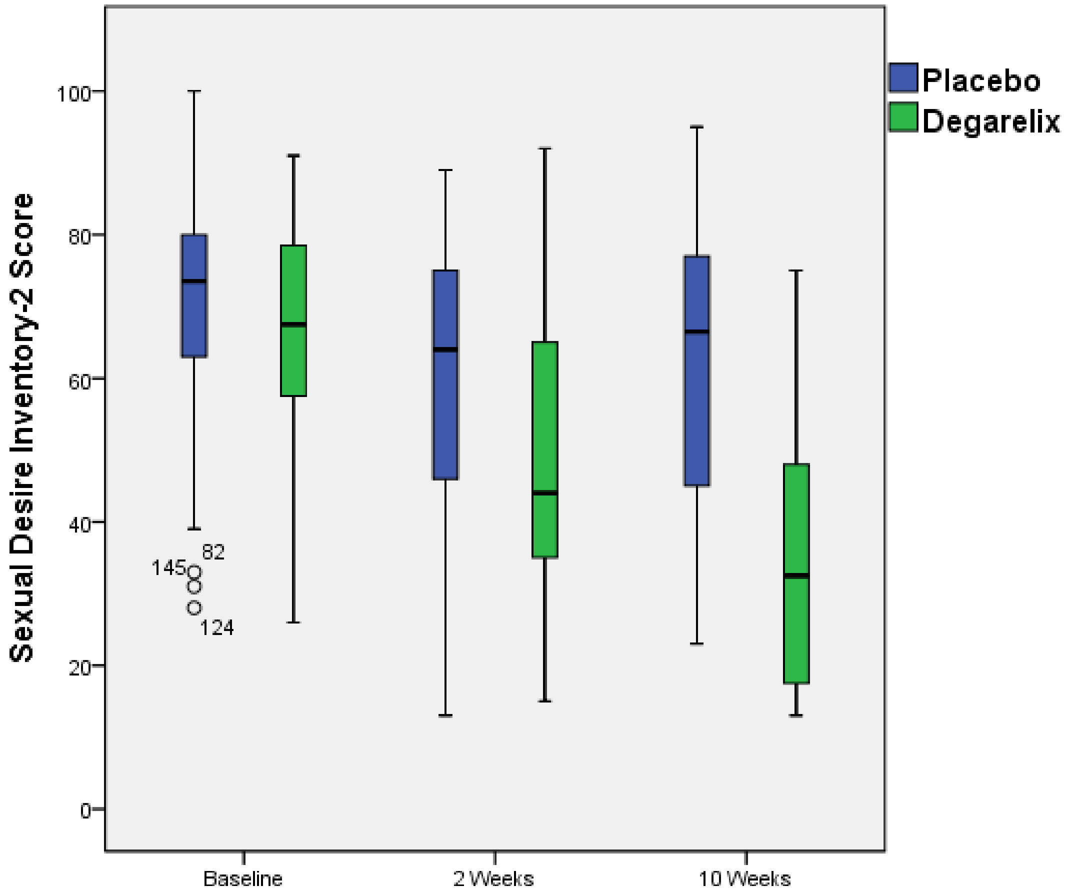

© 2020 Landgren V et al. *JAMA Psychiatry.*

eFigure 4. Box-Plot of Hypersexual Behavior Inventory Score by Treatment Group and Timepoint

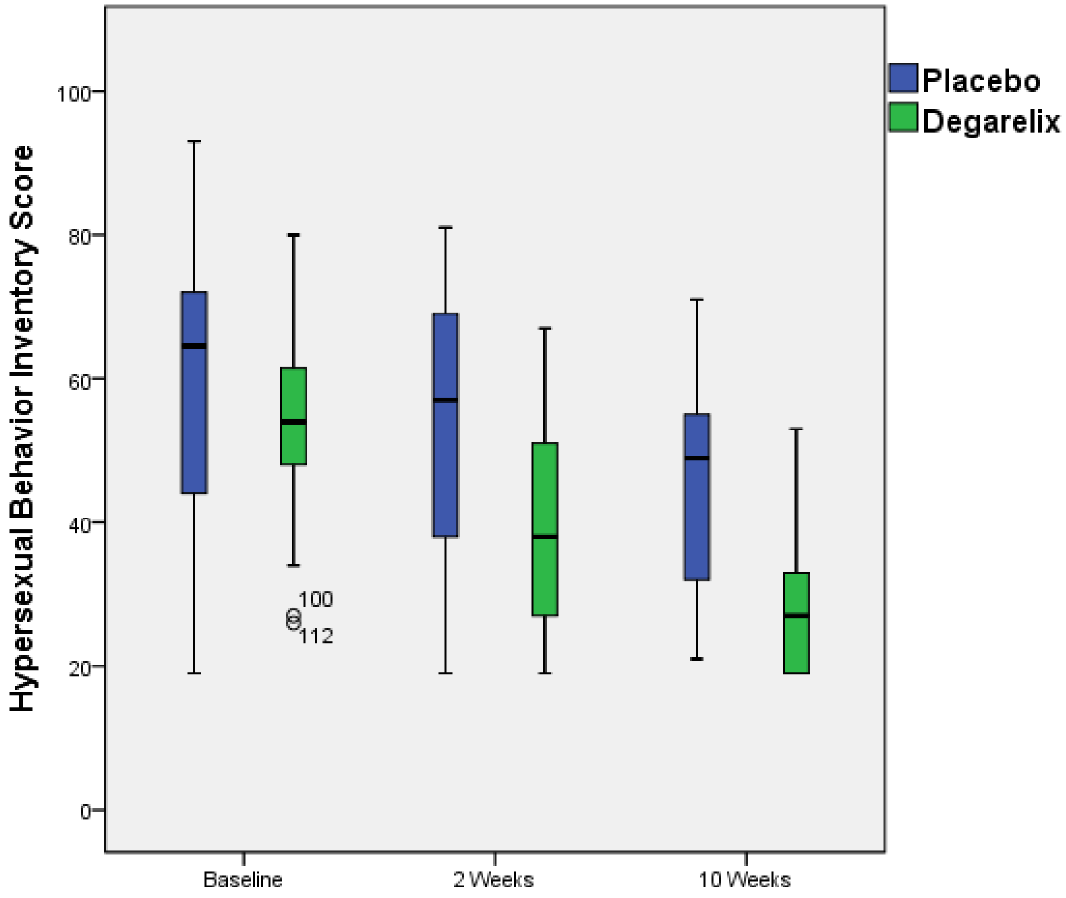

© 2020 Landgren V et al. *JAMA Psychiatry.*

eTable 1. Additional Baseline Psychiatric Characteristics of Participants*

**Outcome measure**		Degarelix (n=26)	Placebo (n=26)
**MINI Neuropsychiatric Interview**			
Depression			
	Current	7 (27)	12 (46)
	Previous	1 (4)	0 (0)
	Now and previous	2 (8)	4 (15)
Dysthymia		3 (12)	5 (19)
Suicide Risk			
	Low	3 (12)	10 (38)
	Moderate	7 (27)	3 (12)
	High	2 (8)	0 (0)
Previous Manic Episode		0 (0)	1 (4)
Hypomania			
	Current	1 (4)	0 (0)
	Previous	0 (0)	1 (4)
Panic Disorder			
	Current	2 (8)	2 (8)
	Previous	2 (8)	0 (0)
Social phobia		6 (23)	9 (35)
Obsessive Compulsive Disorder with non-sexual theme		3 (12)	5 (19)
Post-Traumatic Stress Disorder		0 (0)	0 (0)
Sexually exploited as child*		2 (8)	2 (8)
Psychotic symptoms		0 (0)	2 (8)
Anorexia Nervosa		0 (0)	0 (0)
Bulimia Nervosa		0 (0)	0 (0)
Generalized anxiety disorder		4 (15)	4 (15)
Antisocial Personality Disorder			
	Now and previous	3 (12)	5 (19)
	Now	2 (8)	3 (12)
	Previous	2 (8)	1 (4)
Any disorder according to MINI		17 (65)	24 (92)
Static-99R score – median (range)**		1 (-1 to 8)	1 (-3 to 6)
ASRS-screen positive		7 (27)	11 (42)

© 2020 Landgren V et al. *JAMA Psychiatry.*

RAADS-14 ≥ 22 points		10 (38)	7 (27)
Any disorder according to MINI ASRS or RAADS-14		18 (69)	25 (96)
DUDIT≥3		2 (8)	8 (31)
AUDIT ≥8		2 (8)	4 (15)

* Based on the MINI neuropsychiatric interview (MINI), Static-99R[29(p99)], Adult attention deficit hyperactivity disorder (ADHD) self-report scale (ASRS),[30] Ritvo Autism and Asperger Diagnostic Screening tool (RAADS-14)[13] and Alcohol and Drug Use Disorder Identification Test (AUDIT and DUDIT).[31,32] Participants were asked about experience of childhood sexual abuse as defined by WHO,[33] a question not part of the MINI interview.

** Static-99 is developed and validated for assessment of static risk factors in convicted subjects, i.e. it cannot be reliably interpreted in this patient cohort.

© 2020 Landgren V et al. *JAMA Psychiatry.*

eTable 2. Adverse Events*

**Outcome – no. (%)**	Degarelix (n=25)		Placebo (n=26)	
Visit	2 weeks	10 weeks	2 weeks	10 weeks
No adverse events	2 (8)	9 (36)	16 (64)	19 (73)
Any mild to moderate adverse events				
1-2 adverse events	21 (84)	12 (48)	7 (28)	6 (23)
3-4 adverse events	2 (8)	6 (24)	3 (12)	3 (12)
**General**				
Injection site reactions	22 (88)	0 (0)	1 (4)	0 (0)
Hot flush	0 (0)	6 (24)	2 (8)	0 (0)
Fatigue	3 (12)	2 (8)	3 (12)	1 (4)
Hyperhidrosis	2 (8)	5 (20)	3 (12)	1 (4)
Decreased physical capacity	1 (4)	2 (8)	-	-
Other†	2 (8)	1 (4)	3 (12)	1 (4)
**Gastrointestinal**				
Abdominal discomfort	1 (4)	1 (4)	1 (4)	1 (4)
Nausea or vomiting	3 (12)	1 (4)	1 (4)	0 (0)
**Psychiatric‡**	2 (8)	2 (8)	3 (12)	3 (12)
**Other§**	- (0)	2 (8)	2 (8)	3 (12)
**Serious adverse events**				
Hospital admission due to suicidal ideation	0 (0)	2 (8)	-	-

© 2020 Landgren V et al. *JAMA Psychiatry.*

* Mild adverse events have no impact on participants' health or function. Moderate adverse events have no impact on health but may impact function. Serious adverse events severely impact function or threatens health. One participant assigned degarelix did not receive the injection and is therefore excluded from analysis. Adverse events are coded according to MedDRA classification system.

† Other general disorders included in the degarelix group malaise (1) and headache (1) reported at 2 weeks, and malaise (1) at 10 weeks. In the placebo group pyrexia (1) and headache (2) was reported at 2 weeks and headache (1) at 10 weeks.

‡Psychiatric disorders included in the degarelix group insomnia (2) reported at 2 weeks and insomnia (2) at 10 weeks. In the placebo group disturbance in attention (1), depressed mood (1) and mood swings (1) was reported at 2 weeks and at 10 weeks depressed mood (1), restlessness (1) and nervousness (1).

§ Other adverse events reported at 10 weeks in the degarelix group were dizziness (1) and painful erection (1). In the placebo group syncope (1) and facial rash (1) was reported at 2 weeks, and weight gain (1), polyuria (1) and testis discomfort (1) at 10 weeks.

© 2020 Landgren V et al. *JAMA Psychiatry.*

eTable 3. Blood Sample Abnormalities

**Participants with metabolic measures outside reference range compared to baseline***	Degarelix (n=25)		Placebo (n=26)	
	2 weeks	10 weeks	2 weeks	10 weeks
**Glucose metabolism – no. (%)**				
Fasting glucose	2 (8)	2 (8)	2 (8)	2 (8)
Fasting insulin	2 (8)	3 (12)	4 (15)	6 (23)
Glycated hemoglobin	0 (0)	1 (4)	1 (4)	0 (0)
**Electrolytes – no. (%)**				
Plasma Calcium	3 (12)	3 (12)	1 (4)	1 (4)
Plasma Sodium	1 (4)	1 (4)	1 (4)	1 (4)
Plasma Potassium	1 (4)	1 (4)	2 (8)	0 (0)
**Hepatic and biliary enzymes† -no. (%)**				
Aspartate aminotransferase	4 (16)	5 (20)	0 (0)	1 (4)
Alanine aminotransferase	3 (12)	10 (40)	0 (0)	1 (4)
Gamma-glutamyl transferase	-	-	-	-
Alkaline phosphatase	0 (0)	1 (4)	-	-

* To examine treatment emergent effects, participants with measures outside the reference range both at baseline and follow-up visits were excluded. Except for two cases of plasma potassium and two cases of plasma sodium, all abnormalities were elevations from baseline. Because no blood sample abnormality had any impact on patients' function, health or treatment decisions, they were all considered mild.

† All elevations were below 3,5 times the upper reference range.

© 2020 Landgren V et al. *JAMA Psychiatry.*

eTable 4. Blood Sample Measures*

**Outcome**	Degarelix (n=26)			Placebo (n=26)		
	Baseline	2 weeks	10 weeks	Baseline	2 weeks	10 weeks
**Glucose metabolism**						
Fasting Glucose (<6.0 mmol/L)	6.1±1.7	5.7±0.6	5.8±0.6	5.6±0.6	6.0±1.3	5.6±0.3
Fasting Serum Insulin (<25 mIU/L)	13.9±4.3	14.6±5.7	21.1±9.4	12.5±4.0	24.2±15.3	21.3±9.5
Glycated Hemoglobin (<42 mmol/mol)	35.2±4.3	36.5±4.3	37.0±4.3	35.3±4.6	35.2±4.6	34.6±4.2
Insuline-Glucose Quotient	2.5±0.7	2.6±0.8	3.5±1.1	2.3±0.7	3.8±2.1	3.8±1.6
**Electrolytes**						
Plasma Sodium (137-144 mmol/L)	141.5±1.0	141.2±0.9	141.4±1.0	141.6±0.8	140.5±2.2	141.7±0.8
Plasma Potassium (3.5-4.4 mmol/L)	4.0±0.1	4.1±0.1	4.0±0.1	4.0±0.1	4.0±0.1	4.0±0.1
Plasma Calcium (2.15-2.50 mmol/L)	2.41±0.04	2.43±0.04	2.45±0.03	2.42±0.04	2.40±0.04	2.41±0.03
Plasma Creatinine (<100 µmol/L)	83±5.3	81±5.6	74±8.4	88±7.0	87±5.8	88±7.0
**Hepatic enzymes**						
Aspartate Aminotransferase (<0.76 µkat/L)	0.4±0.1	0.6±0.1	0.6±0.1	0.4±0.1	0.4±0.0	0.5±0.1
Alanine Aminotransferase (<1.1 µkat/L)	0.5±0.1	0.8±0.4	1.1±0.4	0.5±0.1	0.5±0.1	0.5±0.1
Gamma-Glutamyl Transferase (<1,4 µkat/L)	0.5±0.2	0.5±0.2	0.6±0.4	0.5±0.2	0.5±0.2	0.6±0.4
Alkaline Phosphatase (<1.9 µkat/L)	1.2±0.2	1.2±0.1	1.2±0.1	1.2±0.2	1.2±0.1	1.2±0.1
**Hormonal measures†**						
Serum Testosterone (8.6-29.0 nmol/L)	16.2±3.3	0.7±0.2	0.6±0.2	15.1±2.7	15.3±3.3	15.2±3.1
Serum Estradiol (37-147 pmol/L)	109.1±23.6	-	-	99.4±7.8	101.9±9.2	96.8±8.8
Serum Sex Hormone Binding Globulin (18-34 nmol/L)	38.5±9.4	38.9±9.9	47.6±12.2	38.4±9.9	38.9±10.4	35.4±9.6
Follicle Stimulating Hormone (1.5-12 U/L)	6.0±3.9	1.2±0.6	0.9±0.4	5.0±1.4	5.3±1.5	5.3±1.5
Luteinizing Hormone (1.7-8.6 U/L)	4.8±1.4	0.6±0.2	0.8±0.3	5.1±1.0	5.8±1.0	5.5±1.0
Prolactin (86-324 mIU/L)	262.6±172.5	180.1±67.0	209.0±98.4	206.8±43.9	230.9±45.7	216.2±37.3

* Parentheses indicate normal range and unit of measurement at Karolinska University Laboratory. Numbers are mean ±SD.

† The use of immunochemical assays resulted in low precision of hormonal test results in the lower range, and results below the reference range must be cautiously interpreted. Estradiol measurements were undetectable for the majority in both groups at baseline, and in only one participant

© 2020 Landgren V et al. *JAMA Psychiatry.*

assigned degarelix at 2 or 10 weeks. Measures of follicle stimulating hormone, luteinizing hormone, and testosterone were due to the low precision of results in the lower range uninterpretable for a substantial number of participants assigned degarelix at 2 or 10 weeks, but not at baseline.

© 2020 Landgren V et al. *JAMA Psychiatry*.

eTable 5. Depressive Symptoms and Suicidality*

**Measures**	**Degarelix**			**Placebo**			**Odds ratio (95% CI)**			**P value**
	Baseline (n=24)	2 weeks (n=25)	10 weeks (n=24)	Baseline (n=26)	2 weeks (n=26)	10 weeks (n=26)	Baseline	2 weeks	10 weeks	
**MINI interview**										
Dysthymia	0.1±0.0	0.0±0.0	0.0±0.0	0.2±0.1	0.0±0.0	0.1±0.1	0.5 (0.1 to 3.2)	2.0 (0.1 to 63.9)	0.9 (0.0 to 20.2)	0.91
Suicide risk	0.9±0.1	0.4±0.1	0.5±0.1	0.6±0.4	0.4±0.1	0.3±0.1	0.3 (-0.1 to 0.7)	-0.3 (-0.5 to 0.1)	-0.1 (-0.5 to 0.3)	0.33
Depression	0.3±0.1	0.3±0.1	0.2±0.1	0.5±0.1	0.4±0.1	0.3±0.1	0.2 (0.0 to 1.8)	2.3 (0.3 to 20.7)	2.0 (0.2 to 19.5)	0.74
**Depression severity**							**Mean difference (95% CI)**			
MADRS-S	26±2	23±2	26±3	24±2	24±2	27±2	2 (-3 to 8)	-3 (-10 to 4)	-4 (-12 to 4)	0.55

*The proportion of the binary endpoints (MINI depression and MINI dysthymia) were estimated with logistic random-effects regression models, and the ordinal variables (MINI suicide risk (ranging from 0 to 3) and MADRS-S score (ranging from 0 to 48) among participants with depression) with linear random-effects regression models.

© 2020 Landgren V et al. *JAMA Psychiatry.*

eTable 6. Numbers and Proportions (%) of Levels Within EQ-5D Dimensions During the Trial

		**Mobility**			**Self-Care**			**Usual Activities**			**Pain**			**Anxiety**		
		No problems	Some problems	Extreme problems	No problems	Some problems	Extreme problems	No problems	Some problems	Extreme problems	No problems	Some problems	Extreme problems	No problems	Some problems	Extreme problems
**Placebo**																
	Baseline	25 (96)	1 (4)		25 (96)	1 (4)	-	18 (69)	4 (15)	3 (12)	17 (65)	9 (35)	0 (0)	3 (12)	20 (77)	2 (8)
	2 Weeks	25 (96)	1 (4)		25 (96)	1 (4)	-	18 (69)	5 19)	3 (12)	18 (69)	8 (31)	0 (0)	6 (23)	15 (58)	5 (19)
	10 Weeks	22 (85)	4(15)		26 (100)	0 (0)	-	18 (69)	7 (27)	1 (4)	19 (73)	7 (27)	0 (0)	5 (19)	18 (69)	3 12)
**Degarelix**																
	Baseline	20 (77)	3 (12)		21 (81)	2 (8)	-	14 (54)	8 (31)	1 (4)	13 (50)	7 (27)	3 (12)	3 (12)	14 (54)	6 (23)
	2 Weeks	22 (85)	2 (8)		24 (92)	0 (0)	-	20 (77)	3 (12)	1 (4)	16 (62)	7 (27)	1 (4)	8 (31)	9 (35)	7 (27)
	10 Weeks	20 (77)	4 (15)		22 (85)	2 (8)	-	18 (69)	5 (19)	1(4)	15 (58)	7 (27)	2 (8)	7 (27)	12 (46)	5 (19)

© 2020 Landgren V et al. *JAMA Psychiatry.*

eReferences

1. Dallal GE. randomization.com.

2. King BE, Allgeier ER. The Sexual Desire Inventory as a measure of sexual motivation in college students. *Psychol Rep*. 2000;86(1):347-350. doi:10.2466/pr0.2000.86.1.347

3. Moyano N, Vallejo-Medina P, Sierra JC. Sexual Desire Inventory: Two or three dimensions? *J Sex Res*. 2017;54(1):105-116. doi:10.1080/00224499.2015.1109581

4. Spector IP, Carey MP, Steinberg L. The sexual desire inventory: development, factor structure, and evidence of reliability. *J Sex Marital Ther*. 1996;22(3):175-190. doi:10.1080/00926239608414655

5. Cartagena-Ramos D, Fuentealba-Torres M, Rebustini F, et al. Systematic review of the psychometric properties of instruments to measure sexual desire. *BMC Med Res Methodol*. 2018;18(1):109. doi:10.1186/s12874-018-0570-2

6. Fisher TD, Davis CM, Yarber WL. *Handbook of Sexuality-Related Measures*. Routledge; 2013.

7. Giargiari TD, Mahaffey AL, Craighead WE, Hutchison KE. Appetitive responses to sexual stimuli are attenuated in individuals with low levels of sexual desire. *Arch Sex Behav*. 2005;34(5):547-556. doi:10.1007/s10508-005-6280-y

8. Ballester-Arnal R, Castro-Calvo J, Gil-Julia B, Giménez-García C, Gil-Llario MD. A validation study of the spanish version of the hypersexual behavior inventory (HBI): Paper-and-pencil versus online administration. *J Sex Marital Ther*. 2019;45(4):283-302. doi:10.1080/0092623X.2018.1518886

9. Bőthe B, Kovács M, Tóth-Király I, et al. The Psychometric properties of the Hypersexual Behavior Inventory using a large-scale nonclinical sample. *J Sex Res*. 2019;56(2):180-190. doi:10.1080/00224499.2018.1494262

10. Reid RC, Garos S, Carpenter BN. Reliability, validity, and psychometric development of the Hypersexual Behavior Inventory in an outpatient sample of men. *Sex Addict Compulsivity*. 2011;18(1):30-51. doi:10.1080/10720162.2011.555709

11. Montgomery-Graham S. Conceptualization and assessment of hypersexual disorder: A systematic review of the literature. *Sex Med Rev*. 2017;5(2):146-162. doi:10.1016/j.sxmr.2016.11.001

12. Andersen LMJ, Näswall K, Manouilenko I, et al. The Swedish version of the Ritvo autism and asperger diagnostic scale: revised (RAADS-R). A validation study of a rating scale for adults. *J Autism Dev Disord*. 2011;41(12):1635-1645. doi:10.1007/s10803-011-1191-3

13. Eriksson JM, Andersen LM, Bejerot S. RAADS-14 Screen: validity of a screening tool for autism spectrum disorder in an adult psychiatric population. *Mol Autism*. 2013;4:49. doi:10.1186/2040-2392-4-49

© 2020 Landgren V et al. *JAMA Psychiatry.*

14. Baghdadli A, Russet F, Mottron L. Measurement properties of screening and diagnostic tools for autism spectrum adults of mean normal intelligence: A systematic review. *Eur Psychiatry*. 2017;44:104-124. doi:10.1016/j.eurpsy.2017.04.009

15. Shaked D, Faulkner LMD, Tolle K, Wendell CR, Waldstein SR, Spencer RJ. Reliability and validity of the Conners' Continuous Performance Test. *Appl Neuropsychol Adult*. 2019;0(0):1-10. doi:10.1080/23279095.2019.1570199

16. Conners CK, Staff MHS, Connelly V, Campbell S, MacLean M, Barnes J. Conners' continuous performance Test II (CPT II v. 5). *Multi-Health Syst Inc*. 2000;29:175–96.

17. Baron-Cohen S, Jolliffe T, Mortimore C, Robertson M. Another advanced test of theory of mind: evidence from very high functioning adults with autism or asperger syndrome. *J Child Psychol Psychiatry*. 1997;38(7):813-822.

18. Baron-Cohen S, Wheelwright S, Hill J, Raste Y, Plumb I. The "Reading the Mind in the Eyes" Test Revised Version: A Study with Normal Adults, and Adults with Asperger Syndrome or High-functioning Autism. *J Child Psychol Psychiatry*. 2001;42(2):241-251. doi:10.1111/1469-7610.00715

19. Fernández-Abascal EG, Cabello R, Fernández-Berrocal P, Baron-Cohen S. Test-retest reliability of the "Reading the Mind in the Eyes" test: a one-year follow-up study. *Mol Autism*. 2013;4(1):33. doi:10.1186/2040-2392-4-33

20. Vellante M, Baron-Cohen S, Melis M, et al. The "Reading the Mind in the Eyes" test: Systematic review of psychometric properties and a validation study in Italy. *Cognit Neuropsychiatry*. 2013;18(4):326-354. doi:10.1080/13546805.2012.721728

21. Chen K-W, Lee S-C, Chiang H-Y, Syu Y-C, Yu X-X, Hsieh C-L. Psychometric properties of three measures assessing advanced theory of mind: Evidence from people with schizophrenia. *Psychiatry Res*. 2017;257:490-496. doi:10.1016/j.psychres.2017.08.026

22. Sheehan DV, Lecrubier Y, Sheehan KH, et al. The Mini-International Neuropsychiatric Interview (M.I.N.I.): The Development and Validation of a Structured Diagnostic Psychiatric Interview for DSM-IV and ICD-10. *J Clin Psychiatry*. 1998;59(suppl 20):22-33.

23. Sheehan D, Lecrubier Y, Harnett Sheehan K, et al. The validity of the Mini International Neuropsychiatric Interview (MINI) according to the SCID-P and its reliability. *Eur Psychiatry*. 1997;12(5):232-241. doi:10.1016/S0924-9338(97)83297-X

24. Wood KL. The medical dictionary for drug regulatory affairs (MEDDRA) project. *Pharmacoepidemiol Drug Saf*. 1994;3(1):7-13. doi:10.1002/pds.2630030105

25. Brazier J. Is the EQ–5D fit for purpose in mental health? *Br J Psychiatry*. 2010;197(5):348-349. doi:10.1192/bjp.bp.110.082453

26. Burström K, Sun S, Gerdtham U-G, et al. Swedish experience-based value sets for EQ-5D health states. *Qual Life Res*. 2014;23(2):431-442. doi:10.1007/s11136-013-0496-4

© 2020 Landgren V et al. *JAMA Psychiatry*.

27. Sandelowski M. Focus on research methods: Whatever happened to qualitative description? *Res Nurs Health*. 2000. doi:10.1002/1098-240x(200008)23:4<334::aid-nur9>3.0.co;2-g

28. Graneheim UH, Lundman B. Qualitative content analysis in nursing research: Concepts, procedures and measures to achieve trustworthiness. *Nurse Educ Today*. 2004. doi:10.1016/j.nedt.2003.10.001

29. Helmus L, Thornton D, Hanson RK, Babchishin KM. Improving the predictive accuracy of Static-99 and Static-2002 with older sex offenders: revised age weights. *Sex Abuse J Res Treat*. 2012;24(1):64-101. doi:10.1177/1079063211409951

30. Kessler RC, Adler L, Ames M, et al. The World Health Organization Adult ADHD Self-Report Scale (ASRS): a short screening scale for use in the general population. *Psychol Med*. 2005;35(2):245-256.

31. Bergman H, Källmén H. Alcohol use among Swedes and a psychometric evaluation of the Alcohol Use Disorders Identification Test. *Alcohol Alcohol*. 2002;37(3):245-251. doi:10.1093/alcalc/37.3.245

32. Berman AH, Bergman H, Palmstierna T, Schlyter F. Evaluation of the Drug Use Disorders Identification Test (DUDIT) in criminal justice and detoxification settings and in a swedish population sample. *Eur Addict Res*. 2005;11(1):22-31. doi:10.1159/000081413

33. Consultation on Child Abuse Prevention (1999: Geneva S, Team WHOV and IP, Research GF for H. Report of the Consultation on Child Abuse Prevention, 29-31 March 1999, WHO, Geneva. 1999.
