# Supplementary material for: Effect of Gonadotropin-Releasing Hormone Antagonist on Risk of Committing Child Sexual Abuse in Men With Pedophilic Disorder: A Randomized Clinical Trial
Source: JAMA Psychiatry. 2020 Apr 29;77(9):1–9. doi: 10.1001/jamapsychiatry.2020.0440 (PMC7191435; doi:10.1001/jamapsychiatry.2020.0440)
Supplement: Supplement 3. — Data Sharing Statement [file jamapsychiatry-e200440-s003.pdf]

Data Sharing Statement

Landgren. Effect of Gonadotropin-Releasing Hormone Antagonist on Risk of Committing Child Sexual Abuse in Men With Pedophilic Disorder. *JAMA Psychiatry*. Published April 29, 2020. 10.1001/jamapsychiatry.2020.0440

Data
Data available: No

Additional Information
Explanation for why data not available: The dataset contains too many sensitive and private kinds of information about the study participants.
